# DGAT1-associated lipid-retinoid dysregulation correlates with metabolic impairment in the RPE of Stargardt disease

**DOI:** 10.1016/j.isci.2026.116727

**Published:** 2026-07-13

**Authors:** Arpita Dave, Eunice Sze Yin Ng, Zhichun Jiang, Jane Hu, Jordan Tatang, Antonio Paniagua, Jeffrey Doeve, Sachin Parikh, Kevin J. Williams, Linsey Stiles, Roxana A. Radu

**Affiliations:** 1 UCLA Jules Stein Eye Institute and Department of Ophthalmology, David Geffen School of Medicine at UCLA, Los Angeles, CA 90095, USA; 2 Department of Biological Chemistry, University of California, Los Angeles, Los Angeles, CA 90095, USA; 3 UCLA Lipidomics Core, University of California, Los Angeles, Los Angeles, CA 90095, USA; 4 Division of Endocrinology, Diabetes, and Hypertension, Department of Medicine, David Geffen School of Medicine at UCLA, Los Angeles, CA 90095, USA; 5 Department of Molecular and Medical Pharmacology, University of California, Los Angeles, Los Angeles, CA 90095, USA

**Keywords:** macular degeneration, Stargardt disease, retinal pigment epithelium, ATP-binding cassette transporter protein, ABCA4, retinoids, lipidomics, mitochondrial dysfunction

## Abstract

Mutations in *ABCA4* cause Stargardt disease (STGD1) by disrupting retinoid handling and retinal pigment epithelium (RPE) homeostasis, yet the metabolic drivers of RPE degeneration remain unclear. Given that photoreceptor health relies heavily on the support of the RPE, loss of the RPE cells is central to STGD1 pathology. In this study, we show that dysregulation of diacylglycerol O-acyltransferase-1 (DGAT1) is associated with disrupted retinoid-lipid metabolism in STGD1 RPE cells, accompanied by excess retinyl esters and neutral lipid accumulation, impaired lipid processing, and reduced mitochondrial activity. These findings implicate DGAT1-mediated lipid remodeling as a contributing factor to RPE dysfunction in *ABCA4*-associated retinopathies.

## Highlights

• PE accumulates in STGD1 RPE, reflecting impaired ABCA4-mediated lipid transport
• DGAT1 upregulation associates with lipid droplet and retinyl ester accumulation
• Mitochondrial dysfunction results in reduced ATP and metabolic reprogramming

## Graphical abstract

**Figure:**
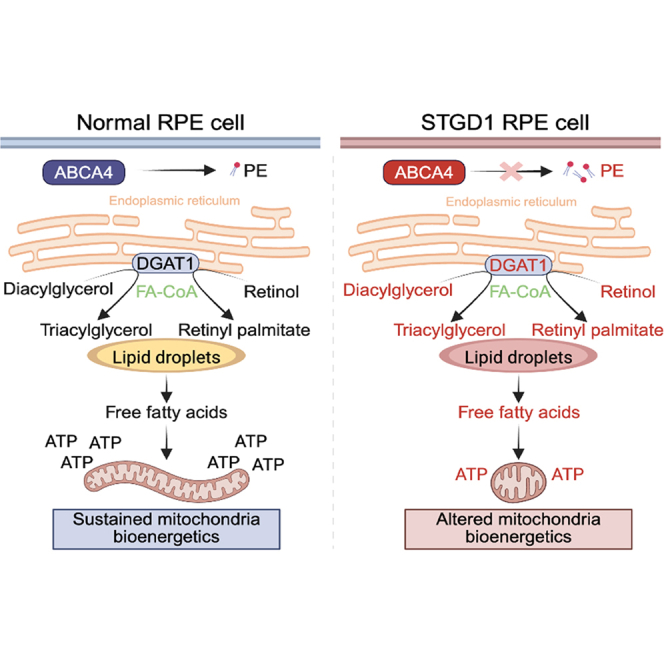

## Teaser

Molecular biology; Neuroscience

## Introduction

Recessive Stargardt disease (STGD1) is a leading cause of inherited macular degeneration caused by mutations in the *ABCA4* gene.^1^ *ABCA4* encodes an ATP-dependent transporter that facilitates the translocation of retinaldehyde-phosphatidylethanolamine (*N*-Ret-PE) and phosphatidylethanolamine (PE) across disc membranes in photoreceptor cells and, at lower levels, in the retinal pigment epithelium (RPE).^2^^,^^3^^,^^4^^,^^5^ Loss of ABCA4 function results in accumulation of toxic bisretinoids, including A2E and its PE-conjugated precursors (A2PE and A2PE-H_2_), which were associated with RPE and photoreceptor degeneration.^5^^,^^6^^,^^7^

Although STGD1 research has historically focused on photoreceptors, where ABCA4 expression is highest, emerging evidence suggests that the low level of ABCA4 in the RPE (∼1% of photoreceptor levels) may nonetheless be functionally significant within specialized membranes microdomains.^3^^,^^5^^,^^8^^,^^9^^,^^10^^,^^11^ Despite more than two decades of investigation, there are currently no Food and Drug Administration (FDA)-approved therapies for STGD1.

The RPE plays a central role in maintaining lipid and retinoid homeostasis by recycling lipids and retinoids derived from daily phagocytosis of photoreceptor outer segments (POSs).^12^ Disruption of this tightly regulated process contributes to lipid accumulation, oxidative stress, and mitochondrial dysfunction, all hallmarks of RPE pathology observed in STGD1 and related maculopathies.^5^^,^^9^^,^^13^^,^^14^^,^^15^^,^^16^^,^^17^ However, the molecular mechanisms linking defective retinoid handling to altered lipid metabolism in the RPE remain incompletely understood.

Here, we identify diacylglycerol acyltransferase 1 (DGAT1), an enzyme that catalyzes the formation of retinyl esters and triacylglycerols from retinol and diacylglycerol, respectively,^18^^,^^19^^,^^20^ as the key mediator of disrupted retinoid-lipid homeostasis in STGD1 RPE cells. Using the *Abca4*^−/−^ mice and well-characterized STGD1 patient-derived induced pluripotent stem cell (iPSC) RPE models, we demonstrate that DGAT1-associated dysregulation is linked to RPE metabolic changes in STGD1. This metabolic imbalance coincides with lipid droplet accumulation and impaired mitochondrial function, suggesting an association between altered lipid storage pathways and RPE degeneration in STGD1.

## Results

### Accumulation of PE lipid class in RPE cells of STGD1 patient

Phosphatidylethanolamine (PE), the second most abundant phospholipid in mammalian cell membranes, plays a key role in endosome formation and membrane fusion.^21^^,^^22^ PE *alone* is also a substrate of ABCA4, flipped across membranes like *N*-Ret-PE.^2^^,^^23^ Abnormal PE membrane distribution in the RPE cells of STGD1(H) patient carrying two disease variants was previously reported, highlighting the biological relevance of ABCA4 in PE recycling.^5^^,^^11^ To quantify PE and its fatty acid species, we performed a direct infusion-tandem mass spectrometry (shotgun lipidomics) of normal and STGD1(H) RPE cells cultured for 2.5 and 7 months in media supplemented with bovine retinal extract, mimicking the physiological presence of retinoids and lipids within the disc membrane of the POS. At 2.5 months, PE levels trended higher in STGD1(H) cells without statistical significance (Figure S1A). By 7 months, total PE levels were ∼2-fold higher in STGD1(H) versus normal RPE cells (Figure S1A), suggesting accumulation over time.

Fatty acid species are known to affect both the structure and function of cell membranes.^21^^,^^24^ PE-linked fatty acid species were consistently elevated in the STGD1(H) versus normal RPE cells (Figures S1B and S1C). At 7 months, PE containing fatty acids FA16:0, FA18:0, FA20:4, and FA22:6 increased ∼1.8- to 2.2-fold compared to normal RPE cells (Figure S1C). These quantitative data align with the *ABCA4* variants specific to STGD1(H), affecting PE-flippase activity. Importantly, the STGD1(H) showed no retinoid accumulation when grown for 6–7 months in culture (Figure S1D), suggesting PE changes as a function of ABCA4 deficiency independent of retinoids and bisretinoid accumulation.

### Accumulation of neutral lipids and lipid droplets in RPE cells of *Abca4*^−/−^ mice and STGD1 patients

**Figure 1:**
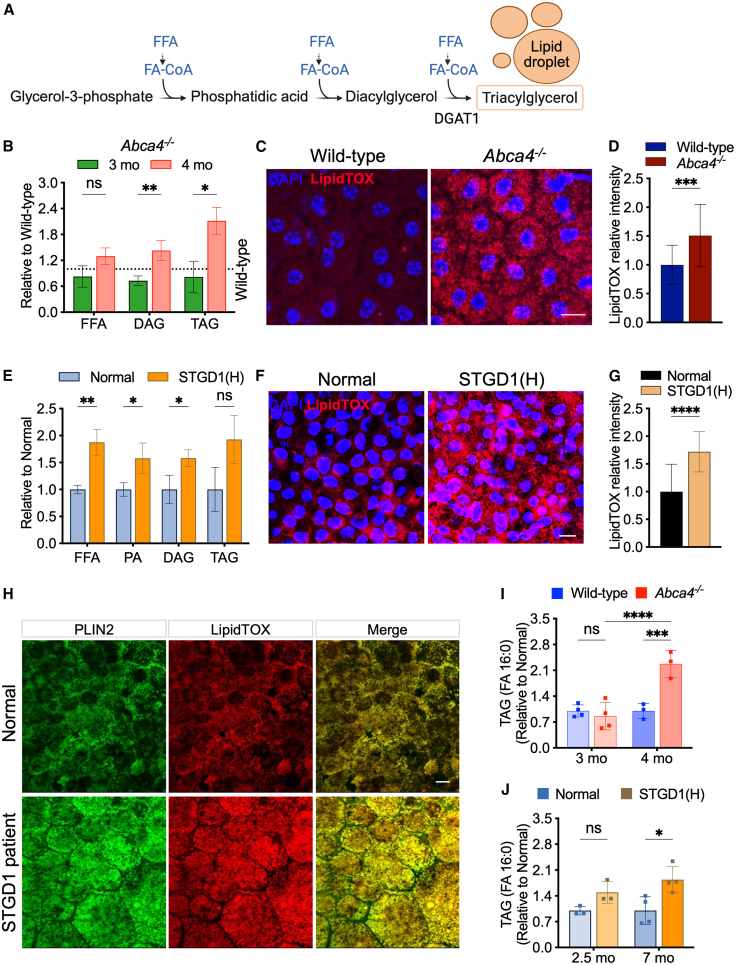
Neutral lipids and lipid droplets in *Abca4*^−/−^ mice and STGD1-patient-derived RPE cells (A) Schematic presentation of triacylglycerol (TAG) synthesis pathway. Free fatty acids (FFAs) are converted to CoA (FA-CoA) and sequentially incorporated into glycerol-3-phosphate, phosphatidic acid (PA), and diacylglycerol (DAG) to generate TAG, which is stored in lipid droplets; DGAT1 is involved in the last step of TAG biogenesis. (B) Lipidomics analysis of FFA, DAG, and TAG levels in RPE cells from 3- and 4-month-old *Abca4*^−/−^ mice, normalized to age-matched 129/Sv wild-type controls (dotted line at 1.0 on the *y* axis); *n* = 3–4 samples, with 6 RPE sheets/n). (C) Representative confocal image of LipidTOX-647 (neutral lipids, red) and DAPI (nuclei, blue) in 7- to 8-month-old mouse RPE flatmounts (scale bars: 10 μm, z stack). (D) Quantification of LipidTOX-647 fluorescence in mouse RPE flatmounts (*n* = 6 RPE flatmounts; 3 mice/genotype BALB/c background; at least 6 images per flatmount). (E) Lipidomics quantification of TAG and the precursors in STGD1(H) and normal iPSC RPE cells cultured for 7 months (*n* = 4 samples, 2 transwells/n). (F) Representative confocal images of LipidTOX-647 (red) and DAPI (nuclei, blue) in iPSC RPE cells cultured for 4 months (scale bars: 10 μm, z stack). (G) Quantification of LipidTOX-647 fluorescence in 4-month iPSC RPE cells (*n* = 3 transwells/genotype; at least 6 images per transwell). (H) Representative confocal images of LipidTOX-647 and PLIN2 staining in the postmortem Stargardt patient (STGD1) and non-Stargardt control (Normal) donor RPE flatmounts (scale bar: 10 μm, middle of z stack). Relative levels of palmitic acid (FA16:0) conjugated to TAG by lipidomics analysis in: (I) RPE from 3- and 4-month-old *Abca4*^−/−^ mice (*n* = 3–4 samples, 6 RPE sheets/n) and (J) STGD1(H)-iPSC RPE cells at 2.5 months and 7 months (*n* = 3–4 samples, 2 transwells/n). Data are presented as mean ± SD. Statistical analyses were performed using unpaired two-tailed *t* tests (with Bonferroni correction) or two-way ANOVA for multiple comparisons; adjusted ∗*p* < 0.05; ∗∗*p* < 0.01; ∗∗∗*p* < 0.001; ∗∗∗∗*p* < 0.0001; ns, not statistically significant.

To compare lipid profiles in the neural retina and RPE, we performed lipidomics on wild-type and *Abca4*^−/−^ mice. At 3 months, retinal and RPE lipid profiles diverged (PC1, 47.3%; PC2, 21.1%) irrespective of their genotypes in principal-component analysis (PCA) (Figure S2A). While overall lipid levels trended lower in the *Abca4*^−/−^ retina and RPE (Figures S2B and S2C), A2E-bisretinoids were previously shown to be elevated at this age.^15^^,^^25^^,^^26^^,^^27^ However, by 4 months, PCA plot showed distinct clustering of RPE of *Abca4*^−/−^ versus wild-type (PC1, 57.8%; PC2, 15.1%) (Figure S2D), with a 2.5-fold triacylglycerol (TAG) species increase (Figure S2E). Within a month, TAG and TAG-intermediates such as free fatty acid (FFA) and diacylglycerol (DAG) rose ∼1.5- to 2.4-fold in the RPE cells of *Abca4*^−/−^ mice (Figures 1A and 1B), indicating progressive accumulation. Furthermore, lipidTOX-647 staining revealed ∼1.5-fold more lipid droplets, a hallmark of aging and disease,^28^ in the *Abca4*^−/−^ mice RPE flatmounts at 7–8 months (Figures 1C and 1D; Figure S3).

STGD1(H) RPE cells showed a similar lipid change, with ∼1.5- to 2.4-fold increase in FFA, lysophosphatidylcholine, phosphatidic acid, and triacylglycerol at 2.5 months, without reaching significance though (Figure S4A). At 7 months, the PCA analysis distinguished STGD1(H) from normal RPE (PC1, 65.8%; PC2, 13.6%) (Figure S4B), with significantly higher levels of several lipid classes (Figure S4C), including FFA, phosphatidic acid, and diacylglycerol (Figure 1E; Figure S4C). LipidTOX-647 staining showed nearly doubled lipid droplet-fluorescence signal in STGD1(H) RPE cells (Figures 1F and 1G; Figure S4D). Additionally, tissue from a postmortem STGD1 donor eye, carrying two *ABCA4* pathogenic variants,^29^ exhibited LipidTOX and Perilipin2 (PLIN2)^30^ colocalization, reflecting widespread lipid droplet accumulation (Figure 1H; Figure S4E), similar to STGD1(H) patient (Figure 1F) and *Abca4*^−/−^ mouse (Figure 1C). These findings highlight the buildup of neutral lipids and lipid droplets as a shared feature in STGD1 patients and *Abca4*^−/−^ RPE cells.

Lipidomics identified FFA class (33%), phosphatidylethanolamine (27%), and phosphatidylcholine (28%) as major lipid classes in RPE. The levels of FFA16:0 (palmitic acid), FFA18:0 (stearic acid), FFA20:4 (arachidonic acid), and FFA22:6 (docosahexaenoic acid/DHA), the most enriched FFA species, were unaltered in both the retina and RPE samples of 3-month-old mice (Figures S5A–S5D). However, we observed an increasing trend of FFA16:0, FFA18:0, and FFA18:2 species in the 4-month *Abca4*^−/−^ RPE relative to wild type (Figure S5E). Further, the FFA22:6 (DHA) associated with the triacylglycerol (TAG) was about 2-fold higher in the 4-month RPE samples (Figure S5F), while no difference in this lipid was observed in the RPE samples of 3-month mice (Figure S5D), suggesting an impaired DHA recycling with age. Notably, palmitic acid (FA16:0) was the most common saturated fatty acid (FA) species in both mouse and human iPSC RPE (Figures S5A, S5C, S5E, S6A, and S6C). Palmitic acid, a key triacylglycerol precursor, increased ∼2.5-fold in 4-month *Abca4*^−/−^ RPE (Figure 1I). In the case of STGD1(H) RPE cells, we found elevated FFAs with different carbon lengths and saturation across culture duration (Figures S6A and S6C), with FFA16:0 being the most abundant. Remarkably, DHA(FA22:6)-containing triacylglycerols increased 2- to 2.5-fold (Figures S6B and S6D), while levels of palmitate (FA16:0)-conjugated triacylglycerols were 1.5- to 2-fold in 2.5-month and 7-month STGD1(H) cultures, respectively (Figure 1J). These data suggest disrupted fatty acid recycling and lipid metabolism in STGD1 RPE cells.

### Age-dependent accumulation of retinyl palmitate in RPE of *Abca4*^−/−^ mice

**Figure 2:**
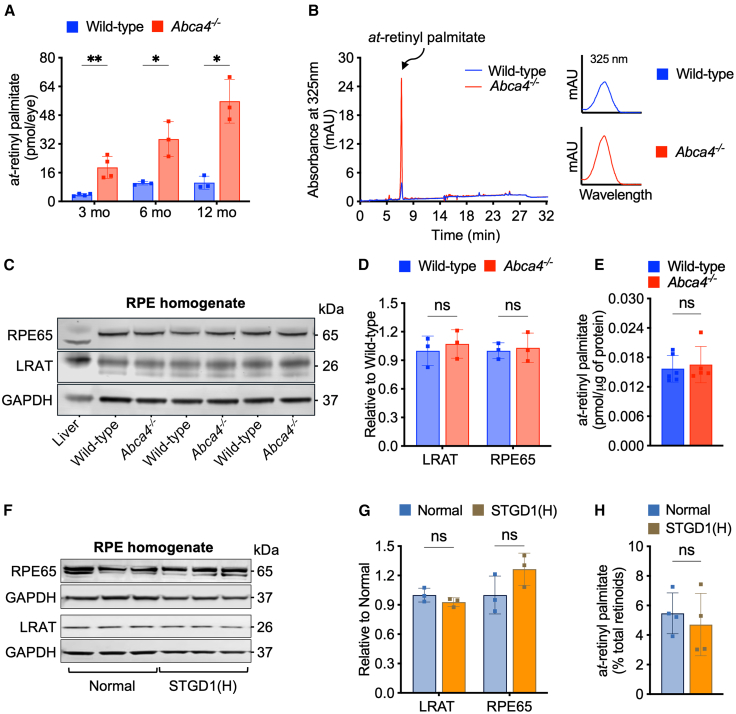
Accumulation of retinyl palmitate in *Abca4*^−/−^ mice and unchanged LRAT/RPE65 protein levels in STGD1 RPE models (A) All-*trans*-retinyl palmitate (a*t*-RP) levels in 3-month (3 mo), 6-month (6 mo), and 12-month (12 mo)-old *Abca4*^−/−^ and wild-type 129/Sv mice RPE/eyecup (pmol; *n* = 3–4, four RPE/eyecup per sample). (B) Representative HPLC chromatogram (325 nm) of 12-month-old *Abca4*^−/−^ (red trace) and wild-type 129/Sv (blue trace) RPE/eyecup hexane extracts (mAU, milli-absorbance unit). Spectra for a*t*-RP peaks at the retention time of ∼7.5 min of chromatograms in (B) are shown on the right. (C) Representative immunoblots of LRAT, RPE65, and GAPDH (glyceraldehyde-3-phosphate dehydrogenase, as internal control) from 12-month-old *Abca4*^−/−^ and wild-type 129/Sv RPE/eyecup (40 μg protein/lane). (D) LRAT and RPE65 protein levels normalized to GAPDH and plotted relative to wild-type levels (*n* = 3 samples, 4 RPE/eyecup per sample, 6 mice/genotype). Experiment repeated three times (6- to 12-month-old mice). (E) LRAT activity measured as a*t*-RP formation after 5 μM retinol incubation (4 min, 37°C) and RPE/eyecup homogenates (6-month-old *Abca4*^−/−^ and wild-type 129/Sv mice), normalized to total protein and corrected for endogenous retinoid levels (pmol//μg protein; *n* = 5–6 samples, 12 mice/genotype). (F) Representative immunoblots of LRAT, RPE65, and GAPDH in 4-month cultured STGD1(H) and normal control iPSC RPE homogenates (15 μg protein/lane). (G) LRAT and RPE65 protein levels normalized to GAPDH and plotted relative to normal iPSC RPE. (H) LRAT activity measured as a*t*-RP formation after 5 μM retinol incubation (4 min, 37°C) with human iPSC RPE homogenates [6- to 7-month-old in culture STGD1(H) patient and normal], presented as percentage of a*t*-RP per total retinoids (a*t*-RP and a*t*-ROL) (*n* = 4, 5 transwells/genotype). Data are presented as mean ± SD. Statistical significance was determined using unpaired two-tailed *t* tests with Bonferroni correction for multiple comparisons; ∗*p* < 0.05; ∗∗*p* < 0.01; ns, not statistically significant.

Palmitate is not only essential for energy storage but also plays a crucial role in retinoid recycling within the RPE cells.^19^^,^^31^^,^^32^ Levels of retinyl palmitate, the predominant form of vitamin A in RPE cells, can be greatly affected by disruptions in intracellular lipid processing and visual cycle dysfunction.^33^ To assess how elevated palmitic acid levels impact retinyl palmitate formation in the *Abca4*^−/−^ RPE, we analyzed retinoids in the eyes of *Abca4*^−/−^ and wild-type mice, at 3-, 6-, and 12-months using high-performance liquid chromatography (HPLC). Levels of all-*trans*-retinyl palmitate (a*t*-RP), the primary retinoid in the dark-adapted RPE/eyecup, accumulated in an age-dependent manner in the *Abca4*^−/−^ mice, surpassing the wild-type level (Figure 2A). Notably, a*t*-RP levels in 12-month-old *Abca4*^−/−^ RPE cells were over 4-fold higher than in wild-type cells (Figures 2A and 2B). To determine whether increased a*t*-RP levels correlate with changes in key RPE visual cycle proteins, we examined lecithin retinol acyltransferase (LRAT), which synthesizes a*t*-RP from retinol,^34^^,^^35^ and RPE65 isomerase (RPE65), which utilizes a*t*-RP as the substrate.^36^^,^^37^^,^^38^ Protein levels of LRAT and RPE65 in 12-month-old *Abca4*^−/−^ RPE/eyecup were comparable to the age-matched wild type (Figures 2C and 2D). Importantly, under optimized assay conditions and substrate availability (5 micro-molar), LRAT enzymatic activity in 6-month-old *Abca4*^−/−^ RPE/eyecup homogenates was indistinguishable from that of age-matched wild type (Figure 2E; Figures S7A–S7C). Similarly, STGD1(H)-patient-derived RPE cells also showed normal LRAT and RPE65 protein levels when cultured for ∼4 months in the presence of bovine retinoid extract in the media (Figures 2F and 2G) and unaltered LRAT enzyme activity when cultured for 6–7 months compared to normal control (Figure 2H; Figure S7D).

### Elevated DGAT1 retinyl ester synthase activity in *Abca4*^−/−^ mice and STGD1-patient-derived RPE cells

In addition to LRAT, DGAT1 functions as an acyl-CoA: retinol acyltransferase in RPE cells, particularly under conditions of elevated retinol availability.^18^^,^^19^ Unlike LRAT, which transfers an acyl group from phosphatidylcholine, DGAT1 utilizes palmitoyl-CoA as an activated FA and operates at a higher *K*m for all-*trans*-retinol (a*t*-ROL); DGAT1 contributes up to 30% of the retinyl palmitate production during light exposure or increased retinol flux in the RPE.^18^^,^^19^ Excess of a*t*-RP was previously linked to retinosomes and intracellular lipid bodies in the mouse RPE.^33^^,^^39^^,^^40^

**Figure 3:**
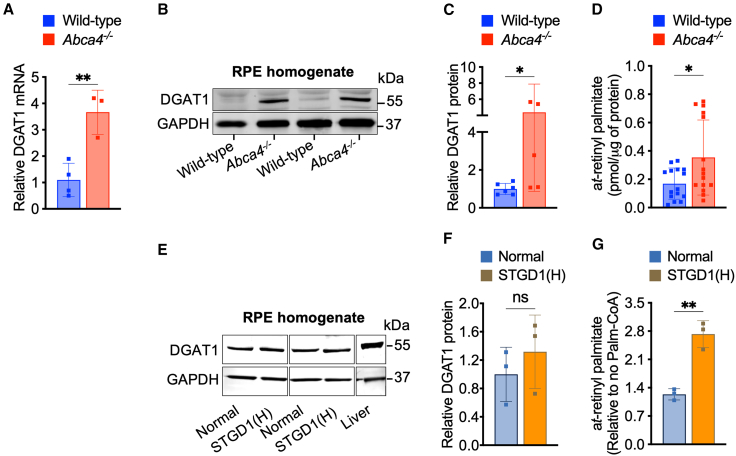
Elevated DGAT1 expression and retinyl ester synthase activity in *Abca4*^−/−^ mice and STGD1-patient-derived RPE cells (A) Relative DGAT1 mRNA levels in 3-month-old *Abca4*^−/−^ and wild-type 129/Sv RPE/eyecup normalized to 18srRNA (*n* = 3–4 samples, 2 RPE/eyecup per sample). Experiment repeated twice with three technical replicates. (B) Representative immunoblots of DGAT1 and GAPDH in 6- to 8-month-old *Abca4*^−/−^ and wild-type (BALB/c) RPE/eyecup homogenates (40 μg protein/lane). (C) DGAT1 protein levels normalized to GAPDH and plotted relative to wild-type (*n* = 6 samples, 4 RPE/eyecup per sample, 6- to 8-month-old 12 mice/genotype). (D) DGAT1 activity measured as palmitoyl-CoA-dependent a*t*-RP formation after 50 μM retinol incubation (30 min, 37°C) in 7-month-old *Abca4*^−/−^ and wild-type (129/Sv) RPE/eyecup homogenates, normalized to protein (pmol/μg protein; mice = 5–7/genotype/experiment). Mouse protein experiments (B–D) were repeated three times. (E) Representative immunoblots of DGAT1 and GAPDH in 4-month cultured STGD1(H) and normal control iPSC RPE homogenates (15 μg protein/lane). (F) DGAT1 protein levels in STGD1(H) iPSC RPE normalized to GAPDH and plotted relative to normal (*n* = 3, 2 transwells/n). (G) DGAT1 activity measured as palmitoyl-CoA-dependent a*t*-RP formation in 3-month cultured STGD1(H) and normal iPSC RPE homogenates, normalized to total protein, and reported as relative to no-palmitoyl-CoA controls (pooled 8 transwells/genotype/experiment). Experiment repeated three times with at least three technical replicates. Data presented as mean ± SD; unpaired two-tail *t* test with Bonferroni correction; ∗*p* < 0.05; ∗∗*p* < 0.01; ns, not statistically significant.

Given the marked a*t*-RP accumulation in *Abca4*^−/−^ RPE despite normal LRAT activity, we next investigated whether DGAT1 contributes to enhanced retinyl ester synthesis. *DGAT1* mRNA levels were approximately 4-fold higher in 3-month-old *Abca4*^−/−^ RPE/eyecup compared to wild-type (Figure 3A), while protein levels were elevated approximately 6-fold at ∼6 to 8 months of age (Figures 3B and 3C). Functionally, using an enzymatic assay with a*t*-ROL (50 micro-molar) as a substrate in the presence of palmitoyl-CoA, we observed ∼2-fold higher palmitoyl-CoA-dependent a*t*-RP production in 7-month-old *Abca4*^−/−^ RPE/eyecup homogenates relative to wild type (Figure 3D).

In STGD1(H)-patient-derived RPE cells, DGAT1 protein levels showed an upward trend at 4 months, although not reaching statistical significance by immunoblot analysis (Figures 3E and 3F). Importantly, enzymatic analysis using substrate-saturating conditions (50 μM retinol and 150 μM palmitoyl-CoA) in 3-month RPE cell homogenates revealed ∼2-fold higher palmitoyl-CoA-dependent at-RP production in STGD1(H) compared to normal controls (Figure 3G), indicating elevated DGAT1 enzymatic capacity. It is noted that these iPSC-RPE cells did not accumulate detectable retinoids under standard culture conditions (Figure S1D), likely reflecting the lower retinoid flux *in vitro* compared to the native retinal environment. The DGAT1 activity assay employed here measures intrinsic enzymatic capacity under defined substrate availability, independent of endogenous retinoid levels. Together with the age-dependent retinyl palmitate accumulation observed in *Abca4*^−/−^ mouse RPE (Figure 2A), where retinoid flux is sustained, these data are consistent with a model in which elevated DGAT1 enzymatic capacity, rather than altered LRAT activity, contributes to excess retinyl ester synthesis in STGD1 RPE when retinoid substrate is available.

### Highly dysregulated metabolic pathways in RPE of *Abca4*^−/−^ mice

**Figure 4:**
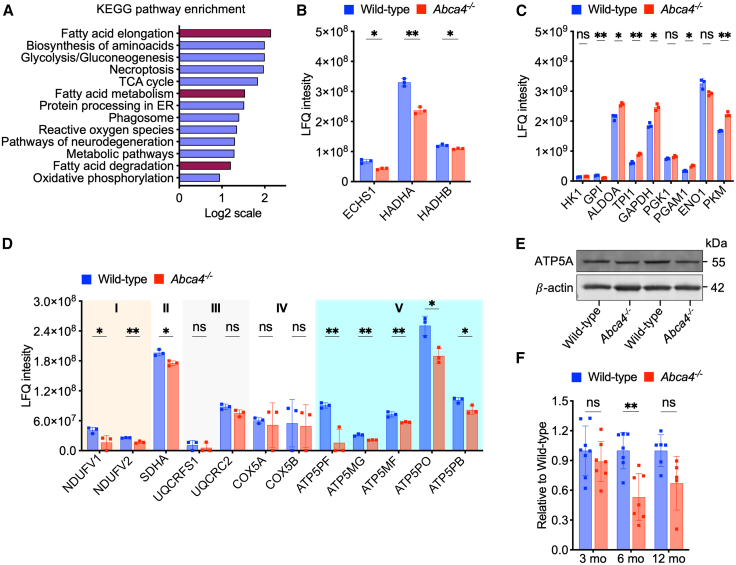
Dysregulated metabolic pathways in *Abca4*^−/−^ mouse RPE Pooled RPE sheets from ∼6- to 7-month-old *Abca4*^−/−^ and wild-type (129/Sv) mice were analyzed by LC-MS/MS proteomics. (A) KEGG pathway enrichment (g:Profiler); lipid-related pathways highlighted in red. (B–D) Label-free quantification (LFQ) of proteins involved in *β*-oxidation pathway proteins (B), glycolysis (C), and mitochondrial oxidative phosphorylation (OXPHOS) complexes I–V (D) (*n* = 3 samples/genotype, 6 RPE sheets/n). (E) Representative immunoblots of ATP synthase (ATP5A) and *β*-actin (internal control) in 6-month-old *Abca4*^−/−^ and wild-type (BALB/c) RPE homogenates (10 μg protein/lane). (F) ATP5A levels normalized to *β*-actin in 3-month (3 mo), 6-month (6 mo), and 12-month (12 mo)-old *Abca4*^−/−^ and wild-type (BALB/c) RPE/eyecup homogenates (*n* = 5–8 samples, 2 RPE/eyecup per n). Data are presented as mean ± SD. Statistical significance was determined using unpaired two-tailed *t* tests with Bonferroni correction for multiple comparisons; ∗*p* < 0.05; ∗∗*p* < 0.01; ns, not statistically significant.

To investigate global protein changes in aging mice, we conducted proteomics analysis of isolated RPE sheets from ∼6- to 7-month-old *Abca4*^−/−^ and wild-type mice using liquid chromatography-mass spectrometry. We identified 213 differentially expressed proteins in *Abca4*^−/−^ RPE, with 99 proteins up-regulated and 114 proteins down-regulated, compared to wild type. Enrichment analysis showed that these proteins were primarily involved in FA metabolism, including elongation and degradation, as well as amino acid biosynthesis, glucose metabolism, necroptosis, reactive oxygen species, phagosomes, pathways of neurodegeneration, and mitochondrial oxidative phosphorylation (OXPHOS) (Figure 4A).

Since phagocytosed POS-derived FAs are a potential source of energy for RPE cellular demands,^32^^,^^41^ and prompted by the top 10 differentially expressed proteins, we analyzed metabolic pathways further. Proteins involved in mitochondrial lipid metabolism by *β*-oxidation were reduced by 15%–30% in the *Abca4*^−/−^ RPE, while most glycolytic proteins were significantly elevated (Figures 4B and 4C). OXPHOS complexes I, II, and V proteins were also markedly reduced (∼20%–80%) in the *Abca4*^−/−^ RPE (Figure 4D; Figure S8A). Although levels of carnitine shuttle proteins CPT1 and CPT2 remained unchanged (Figure S8B), the voltage-dependent anion channel 1 (VDAC1), a gatekeeper for metabolites, anions, and cations across the mitochondrial membranes, was ∼1.8-fold lower in the RPE cells of *Abca4*^−/−^ (Figure S8C).

To address ATP synthesis, we quantified OXPHOS complex V-ATP synthase (ATP5A) in RPE homogenates of 3, 6, and 12 months by immunoblotting (Figures 4E and 4F). ATP5A levels were ∼50% lower in 6- and 12-month-old *Abca4*^−/−^ relative to age-matched wild type (Figure 4F). Cumulatively, our proteomics and lipidomics analyses indicate bioenergetic changes in aging *Abca4*^−/−^ RPE cells.

### Mitochondria functional deficiency in *Abca4*^−/−^ mice and STGD1-patient-derived RPE cells

**Figure 5:**
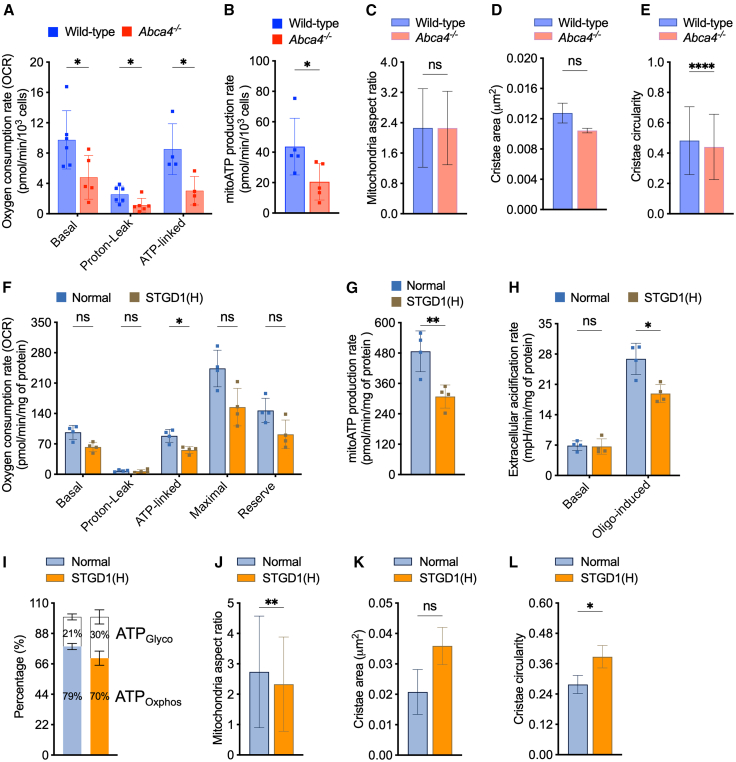
Impaired mitochondrial respiration and altered ultrastructure in *Abca4*^−/−^ and STGD1-patient-derived RPE cells Isolated RPE cells from 10-month-old *Abca4*^−/−^ and wild-type (BALB/c) mice were assayed using a Seahorse HS mini-instrument: (A) basal, proton-leak, and ATP-linked respiration and (B) OXPHOS-derived ATP production rate. Respiration normalized to assay time and cell number. Experiment repeated twice with three replicates (12 mice/genotype). Transmission electron microscopy (TEM) analysis of 6-month-old mouse RPE from *Abca4*^−/−^ and wild-type (BALB/c) mice (*n* = 6 eyes, 3 mice/genotype): (C) mitochondria aspect ratio (190–199 mitochondria for each genotype); (D) cristae area normalized to mitochondria area, and (E) cristae circularity (1,246–1,260 cristae/genotype for D and E). Seahorse analysis of 2-month STGD1(H) and normal iPSC-RPE (day 7 of culture in the assay plate): (F) basal, proton-leak, ATP-linked, maximal respiration, and mitochondrial reserve capacity; (G) OXPHOS-derived ATP production rate; (H) basal and oligomycin-induced glycolysis normalized to protein content; (I) relative contribution of OXPHOS vs. glycolytic ATP production. Experiment repeated three times. TEM of 6-month cultured STGD1(H) and normal iPSC-RPE (3 HA filters/genotype): (J) mitochondria aspect ratio (167–258 mitochondria/genotype), (K) cristae area normalized to the mitochondria area, and (L) cristae circularity (122–278 cristae/genotype for K and L). Data are presented as mean ± SD. Statistical significance was determined using unpaired two-tailed *t* tests with Bonferroni correction for multiple comparisons; adjusted ∗*p* < 0.05; ∗∗*p* < 0.01; ns, not statistically significant.

To better understand the metabolic dysfunction in *Abca4*^−/−^ RPE cells, we measured oxygen consumption rate (OCR) using a Seahorse analyzer in freshly isolated 10-month-old *Abca4*^−/−^ and wild-type RPE cells. Basal respiration, proton leak, and ATP-linked respiration were 2- to 2.6-fold lower in *Abca4*^−/−^ RPE cells (Figure 5A). Consistent with reduced OXPHOS protein levels (Figures 4D–4F), OXPHOS-dependent ATP production in *Abca4*^−/−^ RPE cells was ∼50% of the wild-type levels (Figure 5B), indicating mitochondrial dysfunction.

To confirm these findings in STGD1, we measured OCR and extracellular acidification rate (ECAR) in the patient-derived RPE cells cultured for 2 months. Basal, ATP-linked, maximal, and reserve OCR were lower in STGD1(H) versus normal RPE cells (Figure 5F). Notably, mitochondrial ATP production rate was significantly reduced to ∼70% of the normal levels (Figure 5G). Oligomycin-induced ECAR, indicating glycolytic compensation for ATP synthase inhibition, was ∼40% lower in STGD1 (Figure 5H). In normal RPE cells, ATP production was 79% mitochondrial and 21% glycolytic, whereas in STGD1(H) RPE cells, it shifted to 70% mitochondrial and 30% glycolytic (Figure 5I), aligning with the observed lipid metabolism changes.

### Ultrastructural changes in mitochondria of *Abca4*^−/−^ mice and STGD1-patient-derived RPE cells

To investigate whether mitochondrial dysfunction corresponds to structural changes, we analyzed mitochondrial morphology in a freshly fixed mouse eye (∼6 months old) using transmission electron microscopy (TEM). Measurements of aspect ratio (length-to-width), area, and circularity showed no significant differences from wild-type RPE cells (Figures 5C and 5D; Figures S9A–S9C), suggesting normal fission and fusion processes in *Abca4*^−/−^ RPE cells. However, a slight but significant reduction in cristae circularity (Figure 5E) was observed, in line with decreased mitochondrial respiration and ATP production.

Morphometric parameters were determined for STGD1(H) RPE cells cultured for 6 months. Measurements of STGD1(H) RPE mitochondria showed a reduced aspect ratio and area, while mitochondria circularity and cristae circularity were increased (Figures 5J–5L; Figures S9D–S9F). Differences in mitochondrial morphology and ultrastructure between STGD1(H) patient versus *Abca4*^−/−^ RPE cells may partially reflect culture conditions that do not fully replicate the *in vivo* environment.

### Proposed framework for DGAT1-associated lipid-retinoid dysregulation in STGD1 RPE cells

**Figure 6:**
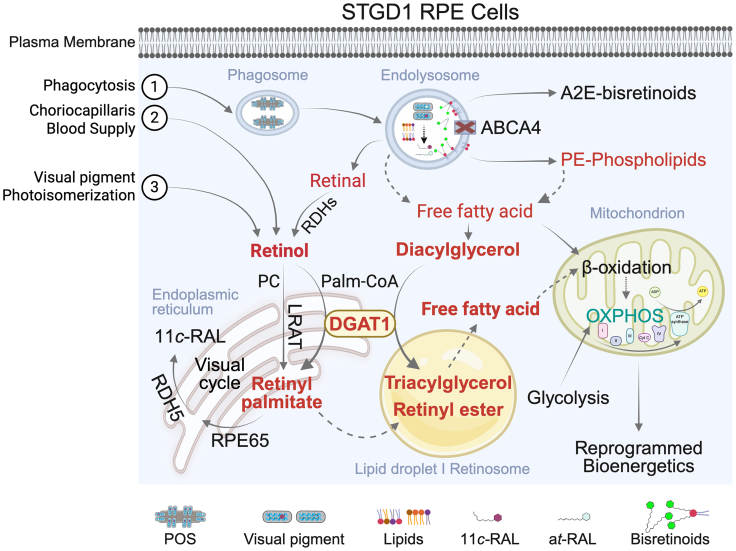
Proposed DGAT1-mediated lipid-retinoid metabolic dysregulation in STGD1 RPE Model summarizing findings. Impaired ABCA4 function in STGD1 RPE leads to accumulation of phosphatidylethanolamine (PE) and bisretinoids (i.e., A2E). Defective endolysosome dynamics promote neutral lipid accumulation (FFA and DAG). Increased DGAT1 activity enhances triacylglycerol (TAG) and all-*trans*-retinyl palmitate (at-RP) synthesis from palmitoyl-CoA and retinol, driving lipid droplet/retinosome formation. Mitochondrial dysfunction, evidenced by reduced *β*-oxidation and OXPHOS and increased glycolysis, is consistent with metabolic reprogramming in STGD1 RPE. Solid arrows indicate pathways supported by data presented in this study. Dashed-style connections within the model represent proposed or inferred relationships that remain to be established by direct experimental intervention. The model is intended as a working hypothesis to guide future investigations rather than a statement of established causality. (created with Biorender.com).

Here, we propose a model in which ABCA4 deficiency drives coordinated lipid and retinoid dysregulation in STGD1 RPE cells (Figure 6). Loss of ABCA4 function leads to progressive accumulation of phosphatidylethanolamine (PE)-retinal adducts and impaired clearance of POSs-derived material within RPE endolysosomes. Disruption of PE homeostasis, along with toxic A2E-bisretinoids, perturbs membrane organization and phagosome-endolysosome dynamics, resulting in defective lipid processing and accumulation of neutral lipids, including free fatty acids and diacylglycerol species. In the RPE, retinol originates from three major sources: (1) POS-derived retinaldehyde released during visual pigment turnover and reduced to retinol by retinaldehyde dehydrogenases (RDHs), with excess retinal also condensing with PE to form A2E-precursors within endolysosomes; (2) basolateral delivery of retinol via retinol-binding protein; and (3) apical transfer of retinol from photoreceptors following photoisomerization. In the context of sustained retinol flux and increased palmitoyl-CoA availability, DGAT1, an endoplasmic reticulum membrane enzyme with dual acyl-transferase activity, is upregulated and becomes the predominant retinyl ester-synthesizing enzyme. Unlike LRAT, whose expression and activity remain unchanged, elevated DGAT1 is associated with enhanced synthesis of both triacylglycerol (TAG) and retinyl ester (such as a*t*-RP), coinciding with excessive lipid droplet and retinosome formation and pathological sequestration of neural lipids and retinyl esters. Accumulation of FFAs, specifically palmitate, further contributes to mitochondrial stress, impaired *β*-oxidation pathway, and reduced oxidative phosphorylation (OXPHOS), and the resulting decline in mitochondrial ATP production coupled with compensatory glycolytic upregulation reflects bioenergetic reprogramming observed in STGD1 RPE cells.

Together, this framework positions DGAT1 as a candidate metabolic node whose elevated expression and activity are associated with lipid droplet expansion, retinyl ester accumulation, mitochondrial impairment, and progressive RPE dysfunction in STGD1. As DGAT1 upregulation is a driver or a compensatory response, upstream lipid dysregulation remains an open question; targeted genetic or pharmacological manipulation of DGAT1 in future *in vivo* studies will be required to establish its causal role.

## Discussion

Retina homeostasis depends on tightly coordinated exchange of retinoids, lipids, and metabolites between the RPE and photoreceptors. Although ABCA4 dysfunction has been primarily linked to bisretinoid accumulation in photoreceptors, the initiating events leading to RPE degeneration in STGD1 remain incompletely defined. Using *Abca4*^−/−^ mice (Table 1) and STGD1-patient-derived RPE cells (Table 2), we identify three interconnected pathological features in STGD1 RPE cells: (1) increased levels of phosphatidylethanolamine, (2) dysregulated diacylglycerol O-acyltransferase-1 (DGAT1) activity causing buildup of triacylglycerols and retinyl esters, within lipid droplets, and (3) impaired mitochondrial bioenergetics.

Lipidomic profiling revealed significant enrichment of PE species in ABCA4-deficient RPE. PE is both a substrate of ABCA4 and the second most abundant phospholipid in mammalian membranes, where it plays critical roles in membrane curvature, fusion, and signaling.^2^^,^^24^ We previously showed genotype-dependent alterations in PE membrane distribution in STGD1 iPSC-RPE cells, accompanied by alkalinization of endolysosomal compartments and impaired Cathepsin D maturation.^5^^,^^11^ Together, these findings support a model in which defective ABCA4-mediated handling of PE-retinal adducts disrupts endolysosomal lipid recycling, leading to progressive alterations in membrane composition. Consistent with this model, both human and mouse STGD1 RPE exhibited excess accumulation of neutral lipids, a phenotype also observed in engineered ABCA4-null iPSC-RPE cells.^42^ It is important to note that the PE elevation reported by Weng et al.^43^ was identified in photoreceptor outer segments; in contrast, the PE accumulation described in the present study was measured by mass spectrometry in isolated RPE sheets and iPSC-derived RPE cells and thus reflects an RPE-autonomous change in ABCA4-deficient cells.

Triacylglycerol (TAG) and FFA, enriched in palmitate and docosahexaenoic acid (DHA) species, were the predominant accumulating lipid classes in STGD1 RPE cells. While lipid droplets buffer excess fatty acids and prevent lipotoxicity, sustained lipid storage reflects an imbalance between synthesis, utilization, and degradation. Given the RPE’s central role in DHA recycling to photoreceptors,^44^ altered neutral lipid handling may have secondary consequences for outer segment renewal. Whether DHA accumulation is protective or deleterious likely depends on metabolic and light conditions, as reported previously.^45^^,^^46^

The intrinsic autofluorescence observed in the 561 nm and 647 nm channels in STGD1 donor eye RPE flatmounts, as well as in the 647 nm channel in *Abca4*^−/−^ mouse RPE flatmounts, co-localizes with LipidTOX and PLIN2 staining, suggesting a contribution from neutral-lipid-containing structures such as lipid droplets. However, bisretinoids, lipofuscin, and oxidized lipid species cannot be excluded as additional sources of this signal. RPE lipofuscin is a heterogeneous mixture of more than 21 fluorescent compounds of lipid and non-lipid origin, and the relative contributions of individual fluorophore classes across different excitation and emission wavelengths remain unresolved.^47^^,^^48^^,^^49^^,^^50^ Far-red autofluorescence in *Abca4*^−/−^ mouse RPE has been attributed to A2E and related bisretinoids^49^; yet lipid peroxidation products, including 4-HNE and MDA, which are elevated in *Abca4*^−/−^ RPE,^15^ are known to generate lipofuscin-like autofluorescence in RPE cells independently of bisretinoid content,^51^ and oxidized lipid species within lipofuscin granules may further broaden the spectral emission profile.^52^ In the present study, the spectral overlap among these fluorophore populations precludes definitive attribution of the observed far-red signal to lipid droplets, lipofuscin, or bisretinoid-containing compartments; wide-spectrum wavelength scanning and spectral unmixing across a larger cohort of well-characterized donor eyes will be required to resolve these contributions in future studies.

The age-dependent increase in all-*trans*-retinyl palmitate in *Abca4*^−/−^ RPE prompted evaluation of retinyl ester synthesis pathways. LRAT and RPE65 protein levels, as well as LRAT enzymatic activity, were unchanged, excluding the canonical visual cycle as the primary cause of retinyl ester accumulation. In contrast, DGAT1 expression and palmitoyl-CoA-dependent retinyl ester synthesis were elevated in both STGD1 models. DGAT1 functions both as diacylglycerol acyltransferase and acyl-CoA:retinol acyltransferase in RPE cells.^18^^,^^19^^,^^34^ Under conditions of increased fatty acyl-CoA and retinol flux derived from photobleaching, POS phagocytosis, and choroidal delivery, DGAT1 may become functionally dominant. Our data are consistent with a shift toward DGAT1-mediated TAG and at-RP synthesis, associated with lipid droplet accumulation and retinoid storage. These findings offer a plausible explanation for the parallel accumulation of neutral lipids and retinyl esters observed in STGD1 RPE, though direct causal evidence awaits further *in vivo* genetic or pharmacological intervention studies.

Neutral lipid accumulation and excess palmitate have been associated with mitochondrial stress in multiple cell types. In aged *Abca4*^−/−^ RPE, we observed reduced levels of β-oxidation and OXPHOS-associated proteins with compensatory upregulation of glycolytic enzymes, a pattern consistent with metabolic reprogramming. Whether palmitate accumulation directly contributes to mitochondrial dysfunction in this context, or whether both are consequences of upstream lipid dysregulation, remains to be determined. Although carnitine shuttle proteins CPT1 and CPT2^53^ were unchanged, reduced VDAC1 abundance and impaired OXPHOX activity support functional mitochondrial compromise, in agreement with prior reports of oxidative stress in ABCA4-deficient RPE cells.^5^^,^^15^ Ultrastructural analyses reveal preserved overall mitochondrial morphology but altered crista architecture, correlating with reduced OXPHOX-dependent ATP production. Because the mitochondrial inner membrane is enriched in PE species, altered PE composition may destabilize respiratory chain organization and super-complex integrity.^54^^,^^55^ Together, the data suggest a functional, rather than a primary structural, mitochondrial defect arising from combined lipid and membrane dysregulation.

Our findings extend the pathophysiology framework of STGD1 beyond bisretinoid toxicity and identify DGAT1-associated lipid-retinoid imbalance as a previously unrecognized feature of RPE dysfunction in ABCA4-deficient cells. Current STGD1 therapeutic strategies, including gene augmentation, vitamin A modulation, and complement-targeted approaches, primarily address upstream or inflammatory components of the disease. Our data suggest that metabolic pathways within the RPE, particularly those governing neutral lipid and retinyl ester synthesis, may also influence disease progression. DGAT enzymes have been implicated in metabolic and lysosomal storage disorders characterized by TAG and retinyl ester accumulation.^56^^,^^57^^,^^58^^,^^59^^,^^60^ While additional *in vivo* validation is required, modulation of DGAT1 activity may represent a rational avenue for further investigation, potentially in combination with retinoid-modulatory therapies.

In summary, this study identifies DGAT1-associated retinyl ester and TAG accumulation as a correlate of ABCA4 deficiency, lipid droplet expansion, and mitochondrial dysfunction in STGD1 RPE and positions DGAT1-mediated lipid remodeling as a candidate contributor to RPE metabolic dysregulation, warranting further mechanistic investigation.

### Limitations of the study

Several limitations should be considered when interpreting our findings. First, the autofluorescence data presented in the Figures S3 and S4E should be interpreted as preliminary and descriptive, as the spectral overlap among lipid droplet-, lipofuscin-, and bisretinoid-associated fluorophores precludes definitive signal attribution at the wavelengths employed (discussed above). Due to the limited availability of genetically confirmed STGD1 donor tissue, quantitative assessment of autofluorescence intensity and spectral characterization across different disease stages was not feasible. Additional studies using larger, well-characterized donor cohorts will be required.

Second, ABCA4 expression in the RPE is substantially lower than in photoreceptors, and the relative contribution of RPE-autonomous pathology to STGD1 progression remains debated. While our data support intrinsic metabolic dysregulation in ABCA4-deficient RPE, this study was not designed to dissect photoreceptor-independent versus photoreceptor-driven mechanisms *in vivo*. Future work using cell-type-specific models will be necessary to resolve these interactions across different ABCA4 genotypes.

Third, human *in vitro* studies were performed utilizing iPSC-derived RPE lines from a single STGD1 patient [STGD1(H)] harboring two ABCA4 variants predicted to produce a non-functional protein. Although this line was selected to parallel the *Abca4*^−/−^ mouse model, this line does not capture the genetic heterogeneity of STGD1. Differences in disease kinetics between *in vivo* mouse RPE and cultured human iPSC RPE likely reflect environmental and metabolic disparities, including lower retinoid flux and the absence of the photoreceptor input *in vitro*. Expanding analysis to additional patient-derived lines representing diverse ABCA4 variants will be important to determine whether DGAT1 dysregulation is genotype-dependent.

Fourth, this study did not directly evaluate complement activation or inflammatory signaling pathways. Lipid species, including oxidized lipids and bisretinoid byproducts, have been implicated in complement modulation in retinal disease; establishing a causal link between DGAT1-driven lipid accumulation and complement pathway activation was beyond the scope of the present work. Dedicated functional studies assessing complement components, inflammatory mediators, and lipid-immune interactions will be required to test this hypothesis.

Finally, as with all iPSC-based systems, epigenetic variation and culture-related artifacts cannot be fully excluded. Nonetheless, findings were validated across biological and technical replicates and corroborated in an independent *Abca4*^−/−^ mouse model, strengthening reproducibility of the core observations. Broader validation across ABCA4 genotypes, longitudinal human samples, and immune-pathway analysis will be necessary to define the full spectrum of disease mechanisms and therapeutic relevance.

**Table 1:** Mouse model

Figure number	Experiments	Age of mice	Genotypes and strains	Number of mice/genotype	Tissue	Number of tissues/genotype
Figure 1	lipidomics	3 mo 4 mo	129/Sv *Abca4*^−/−^	12 9	RPE sheets photoreceptor outer-segments	24 18
Figure 1	immunohistochemistry	7–8 mo	BALB/c *Abca4*^−/−^	3	RPE flatmount	6
Figure 2	retinoids analysis by HPLC	3 mo 6–12 mo	129/Sv *Abca4*^−/−^	8 6 6	RPE/eyecup	16 12 12
Figure 2	immunoblotting	12 mo	129/Sv *Abca4*^−/−^	6	RPE/eyecup	12
Figure 2	enzyme activity	6 mo	129/Sv *Abca4*^−/−^	12	RPE/eyecup	24
Figure 3	gene expression by RT-qPCR	3 mo	129/Sv *Abca4*^−/−^	4	RPE/eyecup	8
Figure 3	immunoblotting	6–8 mo	BALB/c *Abca4*^−/−^	12	RPE/eyecup	24
Figure 3	enzyme activity	7 mo	129/Sv *Abca4*^−/−^	21	RPE/eyecup	42
Figure 4	proteomics	6–7 mo	129/Sv *Abca4*^−/−^	9	RPE sheets	18
Figure 4	immunoblotting	3 mo 6 mo 12 mo	129/Sv *Abca4*^−/−^	8 7 6	RPE/eyecup	16 14 12
Figure 5	mitochondria respiration by Seahorse assay	10 mo	BALB/c *Abca4*^−/−^	12	isolated RPE cells	1.5 × 10^4^ per well (Seahorse plate)
Figure 5	transmission electron microscopy	6 mo	BALB/c *Abca4*^−/−^	3	fixed eye	6

**Table 2:** Human iPSC RPE model

Figure number	Experiments	Age in culture	Genotypes	Number of transwells/genotype
Figure 1	lipidomics	2.5 mo 7 mo	normal STGD1(H)	6 8
Figure 1	immunocytochemistry	4 mo	normal STGD1(H)	3
Figure 2	immunoblotting	4 mo	normal STGD1(H)	6
Figure 2	enzyme activity	6–7 mo	normal STGD1(H)	5
Figure 3	immunoblotting	3 mo	normal STGD1(H)	6
Figure 3	enzyme activity	3 mo	normal STGD1(H)	8
Figure 5	mitochondria respiration by Seahorse assay	2 mo	normal STGD1(H)	2 × 10^4^ cells per well (Seahorse plate)
Figure 5	transmission electron microscopy	6 mo	normal STGD1(H)	3

∼2 × 10^5^ RPE cells/transwell inserts.

## Resource availability

### Lead contact

Further information and requests for resources and reagents should be directed to the lead contact, Roxana A. Radu (radu@jsei.ucla.edu).

### Materials availability

This study did not generate new unique reagents.

### Data and code availability

• Raw and processed datasets are available through Mendeley Data (https://doi.org/10.17632/ssttjz5rhx.1) and may also be obtained upon request from the senior author, Roxana A. Radu.
• This paper does not report original code.
• Any additional information required to reanalyze the data reported in this paper are available from the lead contact upon request.

## Acknowledgments

The authors thank Dr. Michael B. Gorin (UCLA Stein Eye Institute) and STGD1 patient participant under Dr. Gorin’s medical care, without whom this work would not be possible. We acknowledge UCLA undergraduates Sophia Dominguez, Lucineh Kehkejian, Jim Woo, and Ami Pant for their technical assistance with immunoblotting, confocal imaging, and mouse tissue collection. We thank Dr. Sumit Bhutada, Cleveland Clinic, for guiding Dr. Dave for the Proteomics analysis, Dr. Martin-Paul Agbaga, Dean McGee Eye Institute, for scientific insights related to our lipidomics data, and Dr. Elliot Choi, UCLA Stein Eye Institute, for his valuable scientific discussions and critical feedback during manuscript revision. STGD1 donor eye tissue was generously provided by Dr. Vera Bonilha from the Cleveland Clinic. We also thank Dr. Yu Chen, UCLA Proteomics Core, and Dr. Yuekan Jiao, UCLA Stein Eye Institute Imaging Core, for their experimental support. We thank Dr. Joanna Kaylor, Theodore Huynh, and David Stennis-Weatherspoon for their valuable support in performing the DGAT1 enzyme assay. Some reagents were generously provided by Drs. Dean Bok and Gabe Travis (UCLA) and Drs. Phillip Kiser and Kris Palczewski (UCI). The study was supported by the 10.13039/100000053National Eye Institute grant R01 EY025002 (R.A.R.), P30-EY000331 Jules Stein Eye Institute Core Grant for Vision Research, unrestricted grant from 10.13039/100001818Research to Prevent Blindness (RPB, New York), UCLA Eli and Edythe Broad Center of Regenerative Medicine and Stem Cell Research Rose Hills Foundation Innovator Grant (R.A.R.), and the Daljit S. and Elaine Sarkaria Charitable Foundation (R.A.R.). R.A.R. holds the Vernon O. Underwood Family Endowed Chair in Ophthalmology, UCLA David Geffen School of Medicine.

## Author contributions

R.A.R., A.D., and E.S.Y.N. designed the research; A.D., E.S.Y.N., Z.J., J.H., J.D., A.P., K.J.W., L.S., S.P., and R.A.R. performed experiments; A.D., E.S.Y.N., Z.J., J.T., K.J.W., and L.S. analyzed data; A.D., J.H., K.J.W., and R.A.R. generated the figures and illustrations; A.D. and R.A.R. wrote the original draft; all authors reviewed, revised, and approved the final draft; and R.A.R. supervised the complete study.

## Declaration of interests

The authors declare no competing interests.

## STAR★Methods

### Key resources table

**Table:** 

REAGENT or RESOURCE	SOURCE	IDENTIFIER
**Antibodies**		

Goat polyclonal anti-DGAT1	Abcam	Cat# ab59034, RRID: AB_2090799
Rabbit monoclonal anti-DGAT1	Santa Cruz Biotechnology	Cat# sc-271935, RRID: AB_10659239
Total OXPHOS Rodent WB Antibody Cocktail	Abcam	Cat# ab110413, RRID: AB_2629281
Mouse monoclonal anti-LRAT	Generous gift from Dr. Phillip Kiser & Kris Palczewski Labs^34^	N/A
Rabbit polyclonal anti-LRAT	Generous gift from Dr. Dean Bok lab^61^	N/A
Rabbit anti-RPE65	Generous gift from Dr. Gabriel H. Travis lab^36^	N/A
Mouse monoclonal anti- *β*Actin	Thermo Fisher Scientific	Cat# MA5-15452, RRID: AB_11001306
Mouse monoclonal anti-GAPDH	Abcam	Cat# ab8245, RRID: AB_2107448
Rabbit polyclonal anti-Vinculin	Abcam	Cat# ab91459, RRID: AB_2050446
Rabbit polyclonal anti-ADRP/Perilipin 2	Proteintech	Cat# 15294-1-AP, RRID: AB_2878122
Donkey anti-goat IgG Alexa Fluor 647	Thermo Fisher Scientific	Cat# A-21447, RRID: AB_2535864
Alexa Fluor 488 Phalloidin	Thermo Fisher Scientific	Cat# A12379, RRID: AB_3662960
IRDye 680LT Donkey anti-Goat IgG	LI-COR Biosciences	Cat# 925-68024, RRID: AB_2814908
IRDye 800CW Donkey anti-Goat IgG	LI-COR Biosciences	Cat# 926-32214, RRID: AB_621846
IRDye 680LT Donkey anti-Rabbit IgG	LI-COR Biosciences	Cat# 926-68023, RRID: AB_10706167
IRDye 800CW Donkey anti-Rabbit IgG	LI-COR Biosciences	Cat# 926-32213, RRID: AB_621848
IRDye 680RD Donkey anti-Mouse IgG	LI-COR Biosciences	Cat# 926-68072, RRID: AB_10953628
IRDye 800CW Donkey anti-Mouse IgG	LI-COR Biosciences	Cat# 926-32212, RRID: AB_621847

**Biological samples**		

Bovine retinoid extract/Bovine eye	Hu and Bok^62^	N/A
Postmortem RPE flatmounts	generous gift from Dr. Vera L. Bonilha^17^	N/A

**Chemicals, peptides, and recombinant proteins**		

1,4-Dioxane (HPLC grade)	Fisher Scientific	Cat# 60-000-78
16% Paraformaldehyde Aqueous Solution, EM Grade	Fisher Scientific	Cat# 50-980-487
Araldite 502	Electron Microscope Sciences	Cat#10900
Benzonase Nuclease, Purity >90%	Sigma-Aldrich	Cat# 70746-3
Bovine Serum Albumin	Sigma-Aldrich	Cat# A7030
Cacodylate buffer	Electron Microscope Sciences	CAS# 75-60-5
Calcium chloride	Sigma-Aldrich	Cat# C4901
Covergrip	Biotium	Cat# NC0154994
DAPI	Thermo Scientific	Cat# 62248
Dispase II, powder	Gibco	Cat# 17105041
DMEM, high glucose, pyruvate	Gibco	Cat# 11-995-073
DMEM/F-12, GlutaMAX supplement	Gibco	Cat# 10565018
Donkey serum	Sigma-Aldrich	Cat# D9663
DTT (dithiothreitol)	Thermo Scientific	Cat# R0861
Fetal Bovine Serum (FBS)	Sigma-Aldrich	Cat# F4135
Formaldehyde	Electron Microscope Sciences	CAS# 50-00-0
Glutaraldehyde	Electron Microscope Sciences	CAS# 111-30-8
Glycerol	Sigma-Aldrich	Cat# G5516
Goat serum	Sigma-Aldrich	Cat# G9023
Halt Protease Inhibitor Cocktail	Thermo Scientific	Cat# 78429
HCS LipidTOX Deep Red Neutral Lipid Stain	Invitrogen	Cat# H34477
Hexane (HPLC grade)	Fisher Scientific	Cat# H303-4
Human iPSC RPE culturing media, growth factors and reagents	^11^	N/A
Hydroxylamine hydrochloride, 98.0%	Sigma-Aldrich	Cat# 255580
Internal Standard Kit for the Lipidyzer platform	SCIEX (https://sciex.com) Avanti Research	Cat# 5040156 Cat# 330827 Cat# 330830 Cat# 330828 Cat# 791642
iTaq Universal SYBR Green Supermix	Bio-Rad	Cat# 1725121
LICOR Intercept (PBS) Blocking Buffer	LI-COR Biosciences	Cat# 927-70010
ʟ-Glutamine–Penicillin–Streptomycin	Sigma-Aldrich	Cat# G1146
Magnesium chloride	Sigma-Aldrich	Cat# M8266
MEM Non-Essential Amino Acids Solution	Sigma-Aldrich	Cat# M7145
Methanol (HPLC grade)	Fisher Scientific	Cat# AA22909K7
Mouse Laminin	Corning	Cat# CB-40232
Osmium tetroxide	Electron Microscope Sciences	CAS# 20816-12-0
Palmitoyl coenzyme a lithium salt,≥90%	Sigma-Aldrich	Cat# P9716
Propyl gallate, powder	Sigma-Aldrich	Cat# P3130
Propylene oxide	Electron Microscope Sciences	CAS# 75-56-9
Retinol, synthetic, ≥95% (HPLC), crystalline	Sigma-Aldrich	Cat# R7632
Retinyl Palmitate Type IV	Sigma-Aldrich	Cat# R3375
RIPA lysis buffer	Thermo Fisher Scientific	Cat# 89900
Saponin	Sigma-Aldrich	CAS# 8047-15-2
SelexION tuning kit	SCIEX (https://sciex.com)	5040141
Sodium acetate, anhydrous	OmniPur/Millipore	CAS#127-09-3
Tris Hydrochloride	Fisher Scientific	Cat# BP153-500
Trypsin-EDTA (0.25%), phenol red	Gibco	Cat# 25-200-056

**Critical commercial assays**		

Micro BCA Protein Assay Kit	Thermo Scientific	Cat# PI23235
Countess Cell Counting Chamber Slides with Trypan blue	Invitrogen	Cat# C10228
Seahorse XF Cell Mito Stress Test Kit	Agilent	Cat# 103010-100
Absolutely Total RNA Purification Kits	Agilent	Cat# 400800
Superscript III First-Strand Synthesis SuperMix	Invitrogen	Cat#11752050

**Deposited data**		

Mouse RPE: Lipidomics (3 Mo and 4 Mo age)-Raw data	This paper	Mendeley Data https://doi.org/10.17632/ssttjz5rhx.1
Human iPSC RPE: Lipidomics (2.5 Mo and 7 Mo in culture)-Raw data	This paper	
Mouse RPE: Proteomics (6-7 Mo age)-Raw data	This paper	

**Experimental models: Cell lines**		

Human: Normal and Stargardt patient (H) induced pluripotent stem cell derived RPE	Ng et al.^5^^,^^11^; Matynia et al.^8^	UCLA Stein Eye Institute; UCLA Eli and Edythe Broad Center of Regenerative Medicine and Stem Cell Research

**Experimental models: Organisms/strains**		

Mouse: Strain 129/Sv: *Abca4*^−/−^	Weng et al.^43^; Radu et al.^63^	*Abca4*^*tm1Ght*^/J https://www.jax.org/strain/023725
Mouse: Strain BALB/c: *Abca4*^−/−^	Radu et al.^7^^,^^64^	N/A

**Oligonucleotides**		

Mus DGAT1 primers Forward: TCCGTCCAGGGTGGTAGTG Reverse: TGAACAAAGAATCTTGCAGACGA	This paper	Accession # NM_010046
Mus 18srRNA primers Forward: TTTGTTGGTTTTCGGAACTGA Reverse: CGTTTATGGTCGGAACTACGA	This paper	Accession # NR_003278.1

**Software and algorithms**		

Biorender	Biorender	https://www.biorender.com/
ChemStation	Agilent	N/A
Cloud-based Seahorse analytics	Agilent	https://www.agilent.com/en/product/cell-analysis/real-time-cell-metabolic-analysis/xf-software/agilent-seahorse-analytics-787485
Fiji	ImageJ2	https://imagej.net/software/fiji/
Fluoview	Olympus Lifescience	N/A
g:Profiler	University of Tartu, Institute of Computer Science, Bioinformatics, Algorithmics and Data Mining Group BIIT	https://biit.cs.ut.ee/gprofiler/gost
Gatan Microscopy suite	Gatan Inc.	https://www.gatan.com/resources/software
GraphPad Prism 9.0 and 10	GraphPad	https://www.graphpad.com/
Image studio Lite	LI-COR Biosciences	https://www.licor.com/bio/image-studio/
Lipidyzer platform	Sciex	https://sciex.com/support/software-support/software-downloads
Perseus version 1.6.10.0	Max Planck Institute of Biochemistry	https://maxquant.net/perseus/
Proteome Discoverer version 1.4	Thermo Fisher Scientific	https://www.thermofisher.com/order/catalog/product/OPTON-31014?SID=srch-srp-OPTON-31014
Wave 2.6.1	Agilent	https://www.agilent.com/en/product/cell-analysis/real-time-cell-metabolic-analysis/xf-software/seahorse-wave-controller-software-2-6-1-740904

**Other**		

6.5 mm Transwell with 0.4 μm Pore Polyester Membrane Insert, Sterile	Corning	Cat# 07200154
Glass tissue grinder	Wheaton	Cat# 62400493
Immobilon-FL PVDF Transfer Membrane	Millipore Sigma	Cat# IPFL00010
Invitrogen Novex NuPAGE 12% Bis Tris Protein Gels	Thermo Fisher	Cat# NP0341BOX
Invitrogen Novex NuPAGE 4-12% Bis Tris Protein Gel	Thermo Fisher	Cat# NP0321BOX
Millicell Cell Culture Insert, 12 mm, HA mixed cellulose esters, 0.45 μm	Millipore Sigma	Cat# PIHA01250
Precision cover glasses #1.5H thickness	Thor labs	Cat# CG15NH1
Robovials	Thermo	Cat# 10800107
Thermo Superfrost Plus miscroscope slides	Fisher Scientific	Cat# 22037246
Agilent 1100 series HPLC	Agilent Technologies	https://www.agilent.com
EVOM2 Voltohmmeter	World Precision Instruments	https://www.wpiinc.com/var-2754-epithelial-volt-ohm-teer-meter
JEM-1400 Plus transmission electron microscope	JEOL Peabody, MA	https://www.jeol.co.jp
NanoDrop 2000c Spectrophotometer	Thermo Fisher	https://www.fishersci.com/shop/products/nanodrop-2000-2000c-spectrophotometers/ND2000
Odyssey CLx infrared imaging system	LI-COR	https://www.licor.com/bio/imaging-systems
Olympus FluoView FV 1000	Olympus/Evident	https://www.olympus-lifescience.com/en/technology/museum/micro/2004/
PowerTome XL ultramicrotome	RMC Boeckeler	https://boeckeler.com/sample-preparation/ultramicrotomy/powertome/
Real-time PCR	Bio-rad	https://www.bio-rad.com/en-us/product/cfx96-touch-real-time-pcr-detection-system?ID=LJB1YU15
Sciex 5500 with DMS	Sciex	https://sciex.com
Zeiss Axiophot microscope with CoolSNAP digital camera	Zeiss and Media Cybernetics	https://www.zeiss.com

### Experimental model and study participant details

#### Mice

Abca4 knockout mice (*Abca4*^−/−^) were originally generated on 129/Sv and BALB/c backgrounds as described previously,^43^^,^^64^ and have since been maintained as an inbred colony. 129/Sv and BALB/c mice served as age-matched and background controls. An equal number of male and female mice per genotype was included in the experiments. Animals were maintained under a standard 12-hour light/12-hour dark cycle with *ad libitum* access to standard rodent chow and water. *Abca4* knockouts in both strains have been characterized before, and until 9 months show no significant cell loss of RPE and photoreceptors. All procedures adhered to the ARVO Statement for the Use of Animals in Ophthalmic and Vision Research and were approved by the UCLA Institutional Animal Care and Use Committee (IACUC), protocol ARC#2012-057.

#### Human-induced pluripotent stem cells (iPSC) derived RPE culture

Experimental protocols were approved by the UCLA Embryonic Stem Cell Research Oversight and Institutional Review Board (IRB#11-000020 and hPSCRO #2017-008-09), and in accordance with regulations of the Health Insurance Portability and Accountability Act of 1996 (HIPAA), with signed informed consent specifying the use of the information for publications and research presentations. All human samples were de-identified before generating independent iPSC lines for each genotype, before being used in our studies. Human RPE cells were generated from iPSCs derived from a clinically and molecularly characterized male STGD1 patient (STGD1 cell line# SMD5004900HUC) and an unaffected male donor (control, cell line# NDHF). Cell lines were validated for viability, confirmed to be free of mycoplasma contamination, and were karyotyped by the UCLA Broad Stem Cell Core as previously described.^8^^,^^65^ Briefly, iPSCs were maintained in mTESR1 plus to 80% confluency prior to differentiation. RPE differentiation was induced using DMEM/F12 medium with Glutamax supplemented with growth factors.^8^ Emerging RPE patches were manually isolated and passaged twice on mouse laminin-coated plates. RPE cells (P3-P5, 2 × 10^5^ cells) were seeded onto transwell inserts (0.4 μM pore size), mouse-laminin coated HA filters, maintained in Miller medium supplemented with 1-2.5% bovine retinoid extract (BRE) and 5% fetal bovine serum on transwells as described.^5^^,^^8^^,^^11^ Only RPE monolayers exhibiting hexagonal morphology, and transepithelial electrical resistance >200 Ω/cm^2^ were used for experiments. All procedures were conducted in accordance with UCLA Institutional Review Board (IRB) approval and complied with the Health Insurance Portability and Accountability Act of 1996 (HIPAA) guidelines.

### Method details

#### Collection of RPE/eyecup

Mice were euthanized by cervical dislocation, and eyes from *Abca4*^−/−^ and wild-type mice were harvested. In 1X phosphate buffer saline (PBS) (pH 7.4), the anterior segment of the eye was removed, followed by the separation of the neural retina from the eyecup.^3^ RPE/eyecups containing RPE, choroid, and sclera were used for immunoblotting, gene expression study, and enzyme activity analysis.

#### Collection of RPE sheets and photoreceptor outer segments from mice for lipidomics and proteomics

RPE sheets and crude extract of photoreceptor outer segments were collected from 3- and 4-month-old *Abca4*^−/−^ and 129/Sv wild-type mice following published protocols.^11^^,^^62^ RPE sheets represent the RPE cell monolayer devoid of the choroid and sclera parts. Tissues from 6 eyes were pooled together per sample. They were resuspended and homogenized using glass tissue grinders (Wheaton) in 1X PBS on ice. Protein concentrations were measured using the MicroBCA assay kit. The samples were then stored at −80°C for Lipidomics analysis.

#### Collection of human iPSC-derived RPE cells for lipidomics

RPE cells derived from STGD1(H) patient and normal iPSCs were resuspended and dislodged from the HA filters by gentle vortexing in 1X PBS. Cells from two HA filters were pooled together per sample. The number of cells per sample was counted using the trypan blue stain and Countess II system. RPE cells were homogenized using glass tissue grinders. The samples were then stored at −80°C for Lipidomics analysis.

#### Lipid extraction and mass spectrometry-based lipidomics

A modified Bligh and Dyer extraction^66^ was carried out on samples. Prior to biphasic extraction, a 13-lipid subclass Lipidyzer Internal Standard Mix was added to each sample. In follow-up experiments, an internal standard mixture consisting of 70 lipid standards across 17 subclasses was added to each sample. Following two successive extractions, pooled organic layers were dried down in a Thermo SpeedVac SPD300DDA using ramp setting 4 at 35°C for 45 minutes with a total run time of 90 minutes. Lipid samples were resuspended in 1:1 methanol/dichloromethane with 10 mM Ammonium Acetate and transferred to robovials for analysis. Samples were analyzed on the Sciex Lipidyzer Platform for targeted quantitative measurement of 1100 lipid species across 13 classes. In follow-up experiments, an expanded targeted acquisition list consisting of 1450 lipid species across 17 subclasses was employed. The differential Mobility Device on Lipidyzer was tuned with SelexION tuning kit. Instrument settings, tuning settings, and MRM lists are available upon request. Data analysis was performed on an in-house data analysis platform comparable to the Lipidyzer Workflow Manager.^67^ Quantitative values were normalized to micrograms of total protein for mouse samples and cell counts for human iPSC RPE samples. Lipidomics of culture media confirmed the presence of a total 17 lipid classes with an abundance of cholesterol esters, free fatty acids, and phosphatidylcholine.^11^

#### Immunohistochemistry

##### Mouse RPE flatmounts

Eyes from 7-8-month-old *Abca4*^−/−^ and wild-type (BALB/c) mice were harvested and fixed in 4% paraformaldehyde in 1X PBS for 30 min. Under the microscope, the anterior segment was removed, and the posterior segment (neural retina plus RPE/choroid) was cut into four leaflets in 1X PBS. Gently neural retina was removed and RPE flatmounts were quenched with 50 mM NH_4_Cl in 1X PBS for 25 min. Following 1X PBS washes (2 × 5min), they were permeabilized with 0.1% Saponin in 1X PBS for 2 hours, washed 2 × 5min with 1X PBS, and blocked with 1% BSA and 5% goat serum in 1X PBS at 4°C overnight. Next day, flatmounts were incubated with DAPI (1:2000) for 30 min followed by one wash with 1X PBS, and incubation with LipidTOX deep red (1:200) at 4°C overnight. After two washes with 1X PBS, RPE flatmounts were mounted with 5% n-propyl gallate in 100% glycerol on glass slides (1 mm thickness), covered with coverslips and sealed with Covergrip. Images from the peripheral regions were captured with an Olympus FluoView FV1000 confocal microscope using a 60× oil-immersion objective.

##### Human RPE flatmounts

Human donor eye tissues were obtained from the Cole Eye Institute (Cleveland, OH), a generous gift from Dr. Vera L. Bonilha and Foundation fighting Blindness. 5–10 mm^2^ pieces of RPE/Choroid from the peri-macular (PM) fixed in 4% formaldehyde and 0.5% glutaraldehyde in 1X PBS were shipped overnight. On receiving, the tissues were washed with 1X PBS and stored in 2% formaldehyde. RPE-choroid were washed three times and quenched with 50 mM NH_4_Cl and permeabilized with 1% Triton X-100 for 30 min then blocked with 5% goat serum and 1% BSA in 1X PBS at 4°C overnight. The RPE-choroid were exposed to the PLIN2 (1:50) primary antibody for two days at 4°C. After washing, the tissues were incubated with the secondary antibody-conjugated Alexa Fluor dye 594 (1:500) for 1 hour at room temperature. The tissue pieces were then incubated with LipidTOX-647 (1:200) for 30 minutes followed by staining with DAPI nuclear marker (1:1000). The RPE-choroid pieces were mounted with 5% n-propyl gallate in 100% glycerol. Images were captured with an Olympus FluoView FV1000 confocal microscope using a 60× oil-immersion objective.

#### Immunocytochemistry

iPSC-derived RPE cells (2x 10^5^ cells per transwell) were seeded on mouse-laminin coated transwell polycarbonate inserts and grown for 4 months in culture in Miller medium with 2.5% BRE and 5% FBS. Then, the cells with their supporting membrane were fixed in 4% formaldehyde in 1X PBS for 10 min. Cells were washed twice for 5 min and quenched with 50 mM NH_4_Cl for 25 min. They were permeabilized with 0.1% Saponin in 1X PBS for 10 min, washed two times for 5 min, and blocked with 1% BSA and 5% goat serum in 1X PBS for 1 hour. DAPI (1:2,000) was applied for 10 min followed by incubation with LipidTOX deep red (1:200) for 30 min. Cells were mounted with 5% n-propyl gallate in 100% glycerol on glass slides (1 mm thickness) covered with coverslips and sealed with Covergrip. Images were captured with an Olympus FluoView FV1000 confocal microscope using a 60× oil-immersion objective.

#### Confocal microscopy and image analysis

In the confocal microscope, exposure time (voltage), camera aperture, and laser intensities were kept constant across all images. The z-stacks were obtained using the same size of ROI and number of slices for all images in both genotypes. Images from No LipidTOX-stained and LipidTOX-stained slides were captured (at least 6 per slide), and intensities were analyzed on FluoView FV1000 confocal microscope software.

#### RNA extraction and quantitative PCR

RPE/Eyecups from 3-month-old *Abca4*^−/−^ and 129/Sv wild-type mice were collected in RNAlater Tissue Collection solution and were stored at −80°C. Total RNA was extracted using the Absolutely Total RNA Purification kit, and 1 μg of RNA was reverse transcribed using a cDNA synthesis kit. Subsequently, 1 μl of cDNA was amplified (40 cycles), and mRNA levels of DGAT1 and 18S rRNA as an internal control were quantified using a real-time PCR system. The primers utilized are listed in the key resources table. Data analysis was conducted using the 2^−ΔΔCT^ method as described by.^68^

#### Liquid chromatography with mass spectroscopy proteomics analysis

RPE sheets from ∼6-7 months old *Abca4*^−/−^ and 129/Sv wild-type were harvested in 1X PBS with Halt protease inhibitor mixture as described previously and were stored at −80°C. Further, the cells were lysed in cold RIPA lysis buffer by incubation for 5 min and homogenized by three rounds of sonication for 5 seconds, with 1 min of incubation on ice between each round. Protein lysate was separated from cell debris by centrifugation at 14K x *g* for 15 min and was stored at −80°C. Each biological replicate consisted of total protein pooled from 6 eyes in both genotypes.

The protein lysates were subjected to proteomics analysis as mentioned in.^69^ Sequentially, the protein lysates were reduced and alkylated in solution (50 μL) by 5 mM TCEP at 56°C for 1 h, and 40 mM iodoacetamide at RT for 30 min in the dark. Samples were then incubated in 200 μL cold acetone at −20°C for 1 hour and centrifuged at 13K x g for 10 min at 4°C. The supernatant was discarded, and protein pellets were air-dried for 10 min at RT. Next, the proteins were digested by incubating with Trypsin dissolved in 50 mM ammonium bicarbonate at 37°C overnight. Protein digests were then desalted by Empore stage-tip and elutes from the stage-tip were dried by speed vac and re-suspended in 3% acetonitrile with 0.1% formic acid on the following day. 1 μg protein was injected into an ultimate 3,000 nano-LC, which was equipped with a 75 μm × 2 cm trap column packed with C18 3 μm bulk resins (Acclaim PepMap 100) and a 75 μm × 15 cm analytical column with C18 2 μm resins (Acclaim PepMap RSLC). The nano-LC gradient was 3−35% solvent A (A = H_2_O with 0.1% formic acid; B = acetonitrile with 0.1% formic acid) over 40 min and from 35% to 85% solvent B in 5 min at flow rate 300 nL/min. The nano-LC was coupled with a Q Exactive Plus orbitrap mass spectrometer. The ESI voltage was set at 1.9 kV, and the capillary temperature was set at 275°C. Full spectra (m/z 350 - 2000) were acquired in profile mode with resolution 70,000 at m/z 200 with an automated gain control (AGC) target of 3 × 10^6^. The most abundant 15 ions were subjected to fragmentation by higher-energy collisional dissociation (HCD) with normalized collisional energy of 25. MS/MS spectra were acquired in centroid mode with resolution 17,500 at m/z 200. The AGC target for fragment ions were set at 2 × 10^4^ with maximum injection time of 50 milliseconds. Charge states 1, 7, 8, and unassigned were excluded from tandem MS experiments. Dynamic exclusion was set at 45 s.

Raw data was searched against Uniprot mouse database by Proteome Discovered version 1.4 for protein identification. “Precursor Ions Area Detector” node was used within Proteome Discoverer 1.4 for label-free quantification. Following parameters were set precursor mass tolerance ±10 ppm, fragment mass tolerance ±0.02 Th for HCD, up to two mis-cleavages by semi trypsin, methionine oxidation as variable modification, and cysteine carbamidomethylation as static modification. False discovery rate (FDR) was at 1% and minimum of 1 peptide was required for protein identification to yield a total of 1098 identified proteins. The Perseus software (version 1.6.10.0) was utilized for data processing and statistical analysis for the label-free quantitation (LFQ) intensities. Briefly, FASTA identifications and LFQ intensities were uploaded to the ‘Text only’ and ‘Main’ categories respectively on Perseus. Biological samples were grouped into two categories (wild-type and *Abca4*^−/−^). LFQ values were transformed onto Log2 scale. Proteins with less than three valid values in at least one group were filtered out. Differentially expressed proteins between wild-type and *Abca4*^−/−^ was determined using two-sample Student’s *t* test and FDR was controlled with the Benjamini-Hochberg method. Significance was set at FDR <0.05. Proteins that were exclusively detected in one genotype were also subjected to further downstream analysis. Protein enrichment analysis was performed using g:Profiler (version e109_eg56_p17_1d3191d) with the following input parameters: organism – *Mus musculus*, statistical domain scope – only annotated genes, significance threshold – Benjamini-Hochberg FDR <0.05. Fold enrichment was calculated for terms annotated by Kyoto Encyclopedia of Genes and Genomes (KEGG) pathway database using the following equation and graphed with GraphPad prism: *(Number of differential proteins in a pathway ÷ Number of total differential proteins)/(Number of detected proteins in a pathway ÷ Number of total proteins detected)*.

#### Immunoblotting

RPE/eyecups from 3-, 6-, 8- and 12-months old *Abca4*^−/−^ and wild-type mice on BALB/c and 129/Sv backgrounds were collected in 1X PBS with Halt protease inhibitor mixture and sonicated for cell lysis. Cell lysates were incubated with 1X benzonase nuclease for 10 min followed by 1 hour of 0.5% SDS incubation with gentle agitation at room temperature. Protein lysates were separated from cell debris by centrifugation at 1000 g for 5min at 4°C. Total protein concentration was determined using the Micro BCA Protein Assay Kit. 10 μg of total protein was used for ATP5A and *β*-actin, while 40 μg of total protein was used for DGAT1, RPE65, LRAT, GAPDH, and Vinculin protein quantifications. For human iPSC RPE cells, 4-months in culture RPE cells were collected from transwells using cells scraper and vortexing after rinsing the cells with 1X PBS. Cells were then sonicated for 15 cycles on ice in RIPA buffer with protease and phosphatase inhibitors. Lysates were incubated for 1 hour at RT in 1X Benzonase. Supernatant was collected after spinning at 15,000g for 10 min at 4°C. Proteins were separated on either 4–12% or 12% SDS/PAGE gels in MOPS buffer at 100V. Proteins were then transferred on PVDF membranes using a semidry transfer unit at 15V for 1 hour. To block the membranes incubated with Odyssey Blocking Buffer at RT for 1 hour. Membranes were probed with primary antibodies (1:1,000 dilutions) overnight at 4°C and cognate IR dye-labeled secondary antibodies (1:10,000 dilutions) for 1 hour at RT. Membranes were washed with 1X PBS-T (0.1% Tween 20) after primary and secondary antibody incubations. Imaging and analysis were done using the Odyssey CLx Infrared Imaging System and software.

#### LRAT enzyme activity assay

LRAT enzyme activity assay was measured in RPE/eyecup tissue from 6-month-old *Abca4*^−/−^ and 129/Sv wild-type mice as previously described.^70^ For each genotype, 12-14 RPE/eyecups were homogenized in 1x PBS. Each 200 μL reaction contained assay buffer (10 mM Tris pH 8.0, 2 mM CaCl_2_, 2 mM MgCl_2_, 1 mM DTT) (pH8.0), 0.2% BSA, 5 μM all-*trans-*ROL (retinol), and RPE homogenate (∼0.2 mg total protein). Reactions were incubated at 37°C for 4 minutes in a water bath. Control conditions included homogenates incubated for 0 and 4 minutes without substrate, as well as substrate-only (retinol) controls. Reactions were quenched by adding 500 μL ice-cold methanol. Retinoids were extracted with hexane and analyzed by high-performance liquid chromatography (HPLC) using a previously established method.^5^ The same assay conditions were also used for the LRAT assay in human iPSC RPE homogenates.

#### DGAT1 enzyme activity assay

DGAT1-dependent acyl-coenzyme A: retinol acyl-transferase assay was measured in RPE/eyecups from 7-month-old *Abca4*^−/−^ and 129/Sv wild-type mice as previously described.^18^ For each genotype, 10-14 RPE/eyecups were isolated and homogenized in the assay buffer. All buffers and reagents were prepared on the day of the assay. Each 500 μL reaction contained assay buffer (10 mM Tris pH 8.0, 2 mM CaCl_2_, 2 mM MgCl_2_, 1 mM DTT) (pH8.0), 0.2% BSA, 150 μM palmitoyl coenzyme A, 50 μM all-*trans-*ROL (retinol), and RPE homogenate (0.6-1 mg total protein). Reactions were incubated at 37°C for 30 min in amber-colored glass vials in the dark with constant gentle agitation on a rotator. Reactions were stopped by quenching with 500 μL ice-cold methanol. Retinoids were extracted in hexane and analyzed by HPLC using a well-established method.^5^ For DGAT1 activity assay in human iPSC RPE cells, 3-month cultured RPE cells grown in Miller medium with 1% BRE and 5% FBS cultures were used. Cells from 8 polycarbonate transwells were collected and homogenized using a glass homogenizer (15 strokes). The assay was performed as described for the mouse RPE cell homogenate, except here 80-200 μg of total protein was used per reaction. To distinguish background a*t-*RP levels from DGAT1-dependent activity, three control conditions were included: 1. RPE homogenate only (endogenous a*t*-RP content); 2. Reaction mix lacking palmitoyl-CoA (no Palm-CoA); and 3. Substrate only (retinol), reaction mix lacking RPE homogenate.

#### Retinoid extraction and normal-phase liquid chromatography analysis

3-, 6- and 12-month-old *Abca4*^−/−^ and 129/Sv wild-type four eyecups containing RPE were homogenized in 1X PBS buffer containing 200 mM hydroxylamine hydrochloride. Retinoids were extracted from mouse RPE homogenate, LRAT, and DGAT1 assay reaction mix by adding 2 mL of methanol and 2 mL of hexane, followed by centrifugation at 3,000 x g for 5 min. The hexane extraction step was repeated once (2 mL), and the organic phases were pooled upon centrifugation. Organic extract containing retinoids was dried under a stream of nitrogen gas and re-dissolved in 115 μL of hexane. Normal-phase high-performance liquid chromatography (HPLC) was performed to measure a*t*-RP. To confirm the specific peak of a*t*-RP, the spectra were compared with the elution time and peak of the authentic a*t*-RP standards (max abs 325 nm). Sample a*t*-RP peak was quantified by comparing with peak areas of known amounts of authentic a*t*-RP, establishing a calibration curve based on published molar extinction coefficient.^71^ The a*t*-RP pmol were reported per eye in longitudinal retinoids analysis of mouse tissue; for LRAT and DGAT1 enzyme activity assays, a*t*-RP levels were normalized to the total protein amount in each RPE homogenate sample, measured by the BCA protein kit.

#### Isolating and pooling mice RPE cells in HS mini plate

Ten-month-old *Abca4*^−/−^ and BALB/c wild-type mice were used for Seahorse respirometry. Mice were euthanized by decapitation, and eyes were briefly immersed in 70% ethanol before transfer to sterile 1X Hanks’ balanced salt solution without Ca^+2^ and Mg^+2^ (HBSS-A). To minimize cell death during processing, only two eyes were enucleated at a time. Under sterile conditions, the cornea, iris, and lens were removed. The neural retina was separated from the eyecups in 1X HBSS-A. Eyecups were incubated in 2% Dispase (prepared in 1X HBSS-A) at 37°C for 30 min. RPE sheets were carefully isolated in 1X HBSS containing Ca^+2^ and Mg^+2^ and transferred individually into 1 mL Dulbecco’s Modified Eagle Medium (DMEM, high glucose) supplemented with 1X essential amino acids, 10% fetal bovine serum, and 1X Penicillin-streptomycin with L-Glutamine. This procedure was repeated for each mouse to obtain sufficient viable RPE sheets. Collected RPE sheets were pooled and centrifuged at 300 x *g* for 5 min. The pellet was dissociated into a single-cell suspension using 0.25% Trypsin and resuspended in assay medium consisting of DMEM supplemented with 5 mM HEPES, 31 mM NaCl, and 3 mg/L phenol red, 2 mM glutamine, 1 mM pyruvate, and 5 mM glucose (pH 7.4, supplements added fresh on the day of the assay). Viable cells were counted using Trypan blue exclusion on a Countess II cell counter. 1.5 x 10^4^ cells per well were seeded into laminin-coated Seahorse HS mini plate and centrifuged at 200x *g* for 5 min to promote adherence. Cells were then incubated in assay medium for 30 min at 37°C in a non-CO2 incubator before measurement.

#### Isolating and culturing human iPSC RPE cells in XF24 plate

Human iPSC RPE cells (Normal, STGD1(H)) grown for 2 months in Miller medium on HA filters or 6-well plate (passages 3–4) were dissociated with trypsin. A total of 2x10^4^ iPSC RPE cells were seeded onto a laminin-coated XF24 Seahorse plate and cultured for 7 days, with medium changes every 3–4 days. On Day 7, cells were washed twice with assay medium and incubated in assay medium for 30 min at 37°C in the non-CO2 incubator before measurement.

#### Seahorse respirometry and calculations

Mitostress assay was performed to measure oxygen consumption rate (OCR) and extracellular acidification rate (ECAR), indicators of mitochondrial OXPHOS and glycolytic activity, respectively. Basal OCR and ECAR were recorded, followed by sequential injections of 2 μM oligomycin, 0.5 μM carbonyl cyanide-4-(trifluoromethoxy) phenylhydrazone (FCCP), 0.9 μM FCCP, and 2 μM Antimycin A/rotenone. Data were acquired using Wave 2.6.1 software or the cloud-based Seahorse analytics platform. At the end of the assay, mousse RPE cells were fixed and stained with Hoechst dye for nuclear visualization to determine cell number. Respiration rates were normalized to cell number and expressed as pmol/min/10^3^ cells. For human iPSC-RPE cells, total protein was measured using a BCA protein kit, and respiration rates were normalized to protein concentration and expressed as pmol/min/mg of protein. Parameters including basal respiration, proton-leak, spare respiratory capacity, and ATP production rate were calculated as previously described in.^72^^,^^73^

#### Transmission electron microscopy and image analysis (TEM)

6-month-old *Abca4*^−/−^ and BALB/c wild-type mice were anesthetized with isoflurane and perfused with a fixative containing 2% formaldehyde and 2.5% glutaraldehyde in 0.1M phosphate buffer (pH 7.4). Eyes were enucleated and immersed-fixed in freshly prepared 2% formaldehyde and 2% glutaraldehyde in 0.1M cacodylate buffer (pH 7.4) at 4°C. Human iPSC RPE cells (Normal, STGD1 (H)) (passage 3) cultured for 6 months in 1% BRE-containing Miller medium on laminin-coated HA filters were fixed and stored in the same fixative solution.

Fixed mouse eyes and human iPSC RPE cells were washed in 0.1M cacodylate buffer and postfixed with 1% tetraoxide (OsO4, v/v) in ultrapure water for 1 hour at RT in the dark. Samples were then dehydrated through a graded ethanol series, transitioned through propylene oxide, and embedded in Araldite resin (Electron Microscopy Sciences). Ultra-thin sections (70 nm) were cut on a PowerTomeX ultramicrotome, collected on 200-mesh copper grids, and stained with uranyl acetate and lead citrate to enhance contrast. Images were acquired using a JEM-1400 Plus transmission electron microscope equipped with an Orius SC1000A camera at 2,000x to 15,000× magnification and captured using Gatan Microscopy Suite Software.

TEM Images were analyzed using open-source Fiji software (ImageJ). The scale was calibrated according to the image scale bar using the straight-line tool. Measurement parameters (Area, Shape Descriptors, Display Label) were selected under *Analyze* > *Set Measurement.* Regions of interest (ROIs), including mitochondria and cristae area, were manually delineated using ROI Manager. Quantitative measurements were obtained using *Measure* function for each selected ROI.

### Quantification and statistical analysis

Statistical analyses were carried out using GraphPad Prism 10. For comparisons of two or more groups, unpaired t-test, two-tailed t-test, one-way or two-way ANOVA with Bonferroni correction were applied. Statistical details for all experiments, including the specific tests used, exact values of n, and what n represents (e.g., number of animals, RPE sheets, transwells, or cells per well), are provided in the figure legends, the Experimental Timelines (Tables 1 and 2), and the method details of the techniques used.
