# Supplementary material for: DGAT1-associated lipid-retinoid dysregulation correlates with metabolic impairment in the RPE of Stargardt disease
Source: iScience. 2026 Jul 13;29(8):116727. doi: 10.1016/j.isci.2026.116727 (PMC13382123; doi:10.1016/j.isci.2026.116727)
Supplement: Document S1. Figure S1–S9 and Table S1 [file mmc1.pdf]

iScience, Volume *29*

Supplemental information

DGAT1-associated lipid-retinoid dysregulation correlates with metabolic impairment in the RPE of Stargardt disease

Arpita Dave, Eunice Sze Yin Ng, Zhichun Jiang, Jane Hu, Jordan Tatang, Antonio Paniagua, Jeffrey Doeve, Sachin Parikh, Kevin J. Williams, Linsey Stiles, and Roxana A. Radu

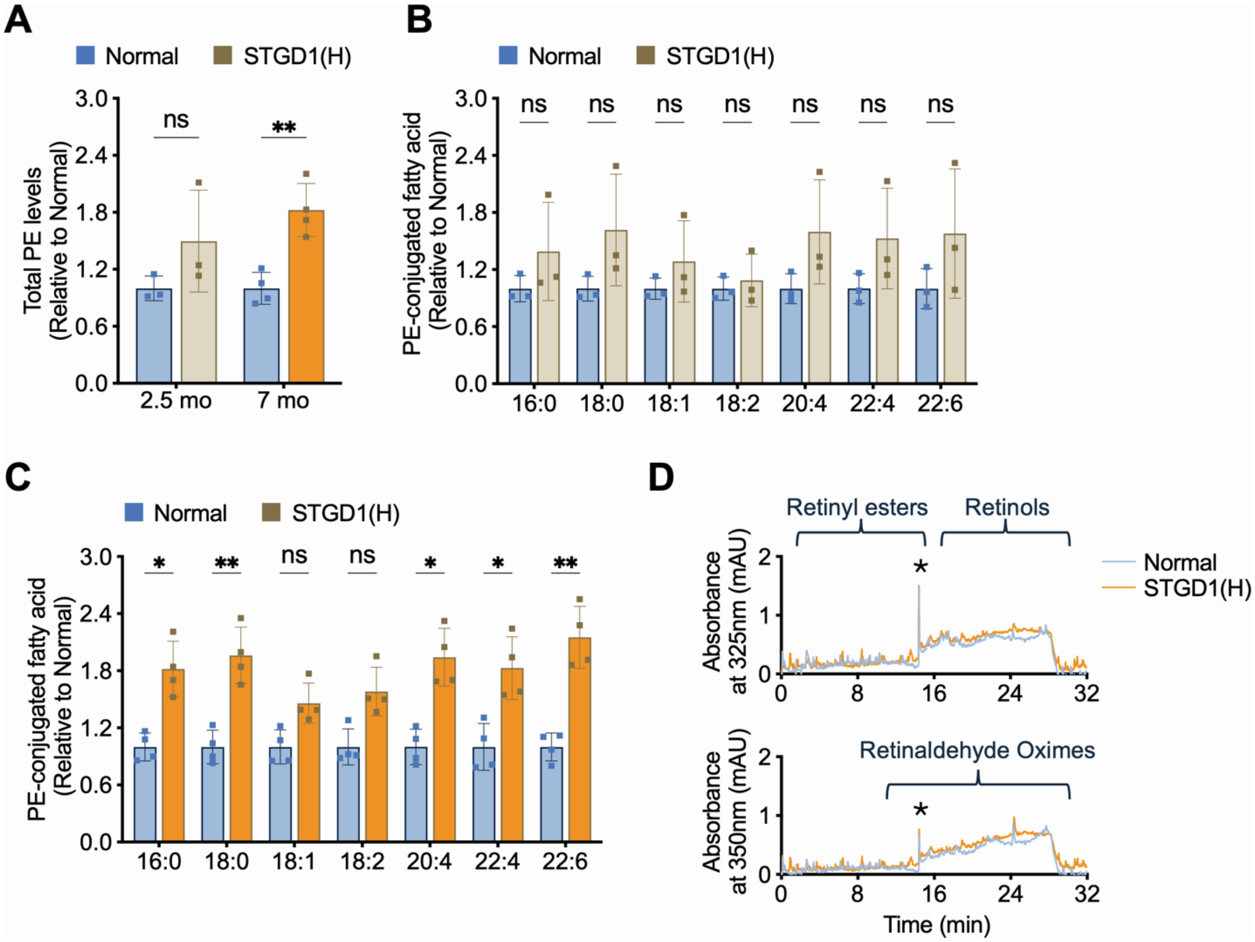

Suppl. Fig. S1 Accumulation of PE lipid class in STGD1 patient RPE cells. STGD1(H) patient and Normal human iPSC RPE cells cultured in Miller medium containing bovine retinoid extract and fetal bovine serum were subjected to Shotgun lipidomics (n=3-4 samples, 2 transwells/n). [**A**] Relative levels of total phosphatidylethanolamine (PE) in STGD1(H) RPE cells cultured for 2.5 months and 7 months, expressed relative to Normal controls. [**B**] Fold changes in PE-fatty acid species in STGD1(H) iPSC RPE cells cultured for 2.5-months. [**C**] Fold changes in levels of PE-fatty acid species in STGD1(H) patient iPSC RPE cells cultured for 7 months. [**D**] Representative HPLC chromatograms of hexane extracts of homogenates from STGD1(H) and Normal RPE cells cultured for 6-7 months, monitored at 325 nm (retinyl esters and retinols) and 350 nm (retinaldehyde oximes, syn and anti), demonstrating absence of detectable retinoids; (*) asterisks indicate changes in solvent gradient. Data are presented as mean ± SD. Statistical significance was determined using unpaired two-tailed *t*-tests with Bonferroni correction for multiple comparisons; adjusted *$p < 0.05$; **$p < 0.01$.

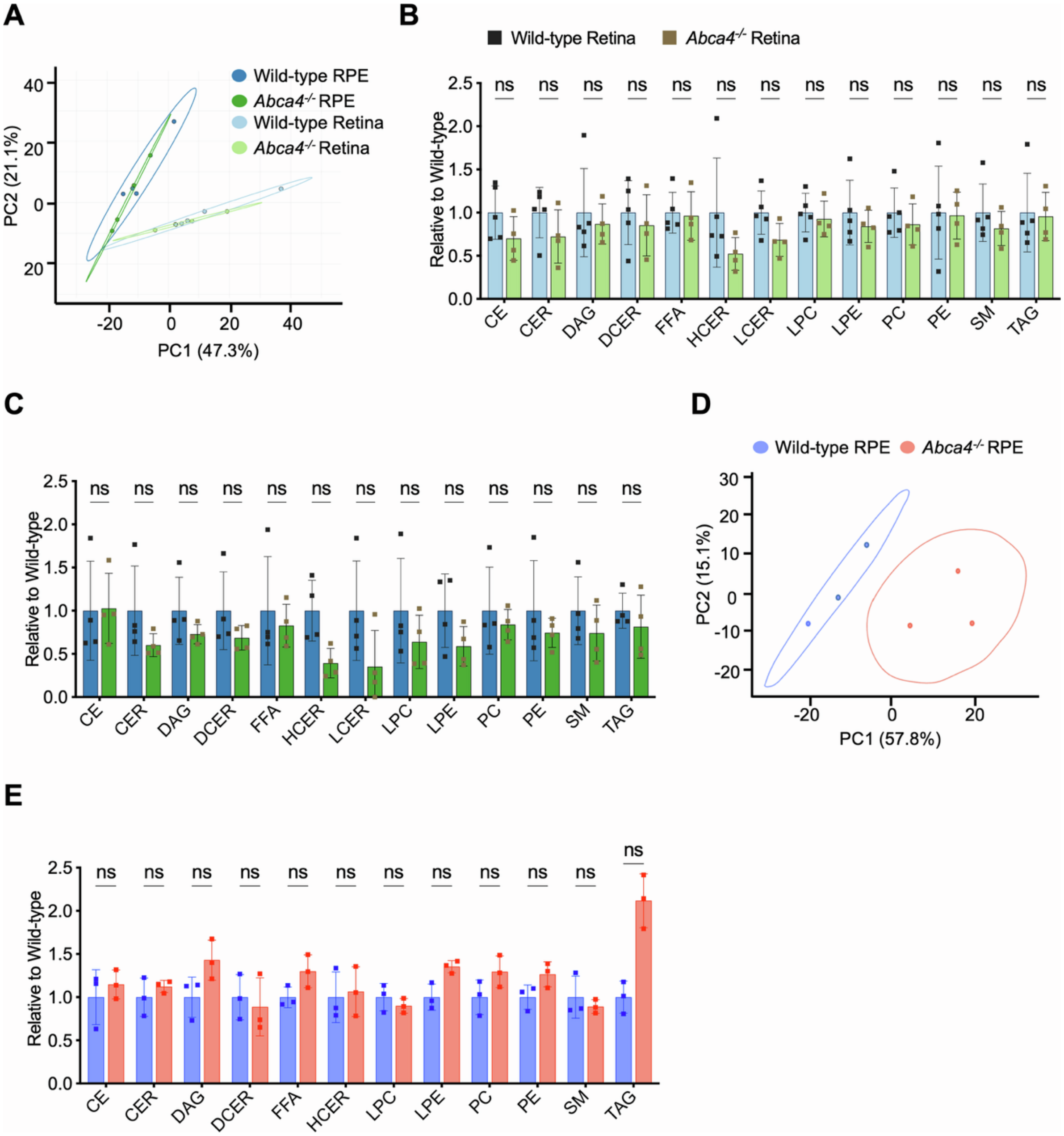

Suppl. Fig. S2 Lipidomics in *Abca4$^{-/-}$* and wild-type mice. [A] Principal component analysis (PCA) plot of lipidomic profiles from retina (enriched in photoreceptor outer segments) and RPE cells collected from 3-month-old wild-type (129/Sv) and *Abca4$^{-/-}$* (129/Sv) mice. **[B]** Lipid class profile of retina from 3-month-old *Abca4$^{-/-}$* mice, expressed relative to wild-type. **[C]** Lipid class profile of RPE from 3-month-old *Abca4$^{-/-}$* mice, expressed relative to wild-type. **[D]** PCA plot of RPE cells collected from 4-month-old wild-type (129/Sv) and *Abca4$^{-/-}$* (129/Sv) mice. **[E]** Lipid class profile of RPE from 4-month-old *Abca4$^{-/-}$* mice, expressed relative to wild-type. Data are

presented as mean ± SD. Statistical significance was determined using unpaired two-tailed *t*-tests with Bonferroni correction for multiple comparisons; *ns* – not statistically significant.

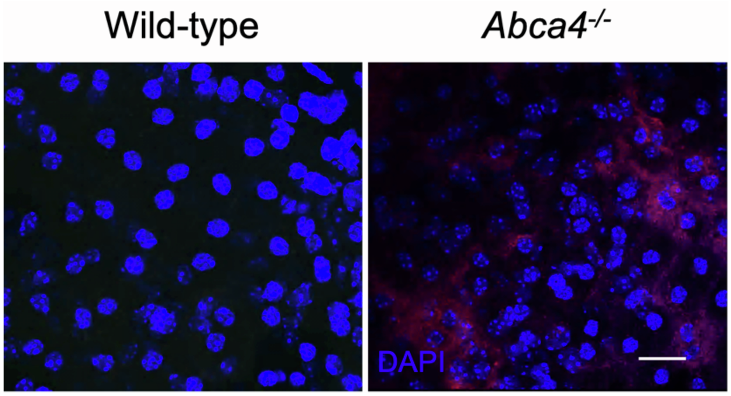

Suppl. Fig. S3 Confocal images for no LipidTOX controls of mouse RPE flatmounts. Control images corresponding to Fig. 1C.

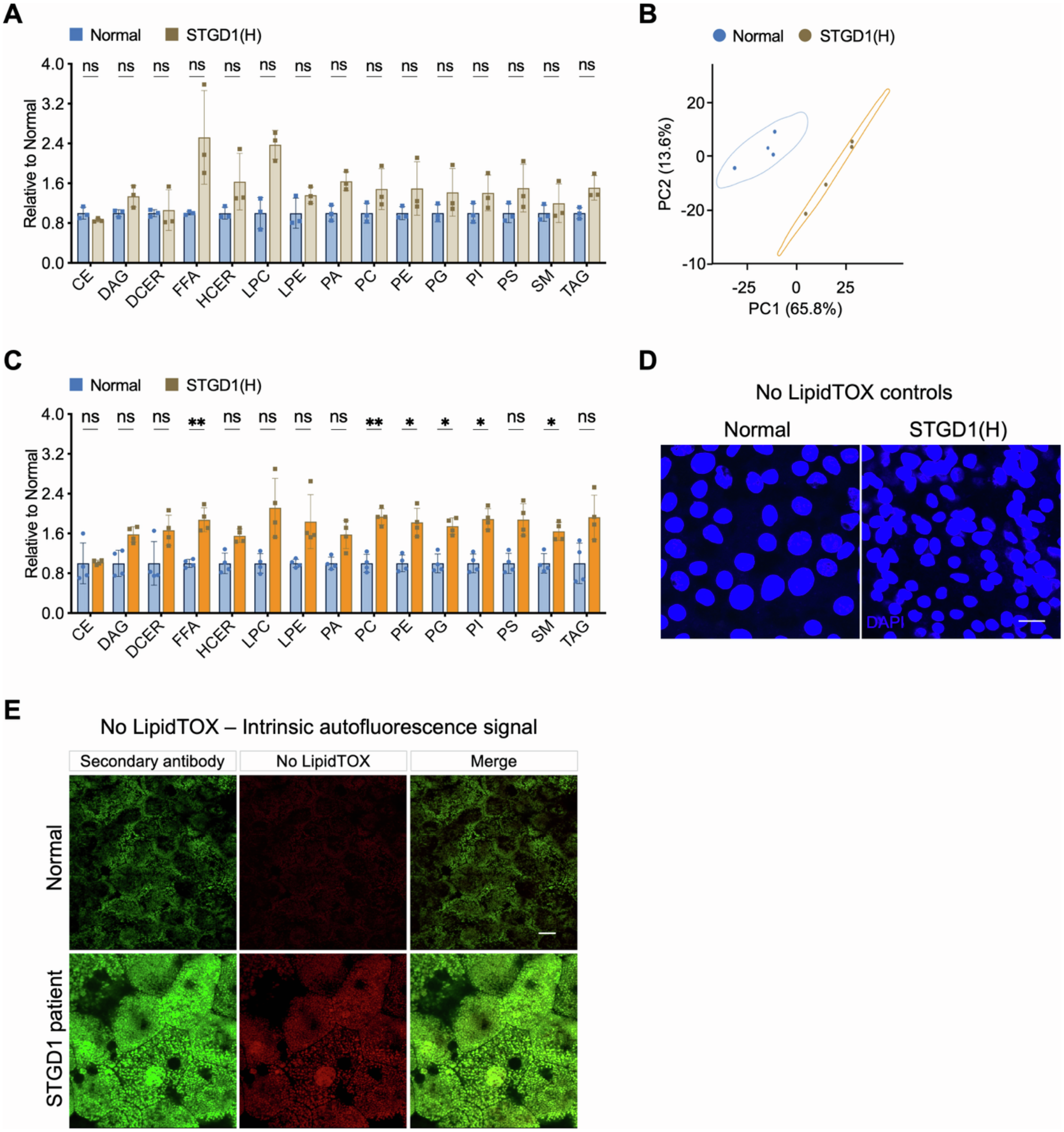

Suppl. Fig. S4 Lipidomics and confocal imaging in STGD1(H) patient versus normal RPE. **[A]** Lipid class profile of STGD1(H) RPE cells cultured for 2.5 months, expressed as relative to Normal control. **[B]** Principal component analysis (PCA) plot of lipidomic profiles from STGD1(H) and Normal iPSC RPE cells cultured for 7 months. **[C]** Lipid class profile of STGD1(H) RPE cells cultured for 7 months, expressed as relative to Normal control. **[D]** Confocal images for no

LipidTOX control corresponding to Fig. 1F. **[E]** Confocal images for no LipidTOX control corresponding to Fig. 1H. Data are presented as mean ± SD. Statistical significance was determined using unpaired two-tailed *t*-tests with Bonferroni correction for multiple comparisons; adjusted *$p < 0.05$; **$p < 0.01$; *ns* – not statistically significant.

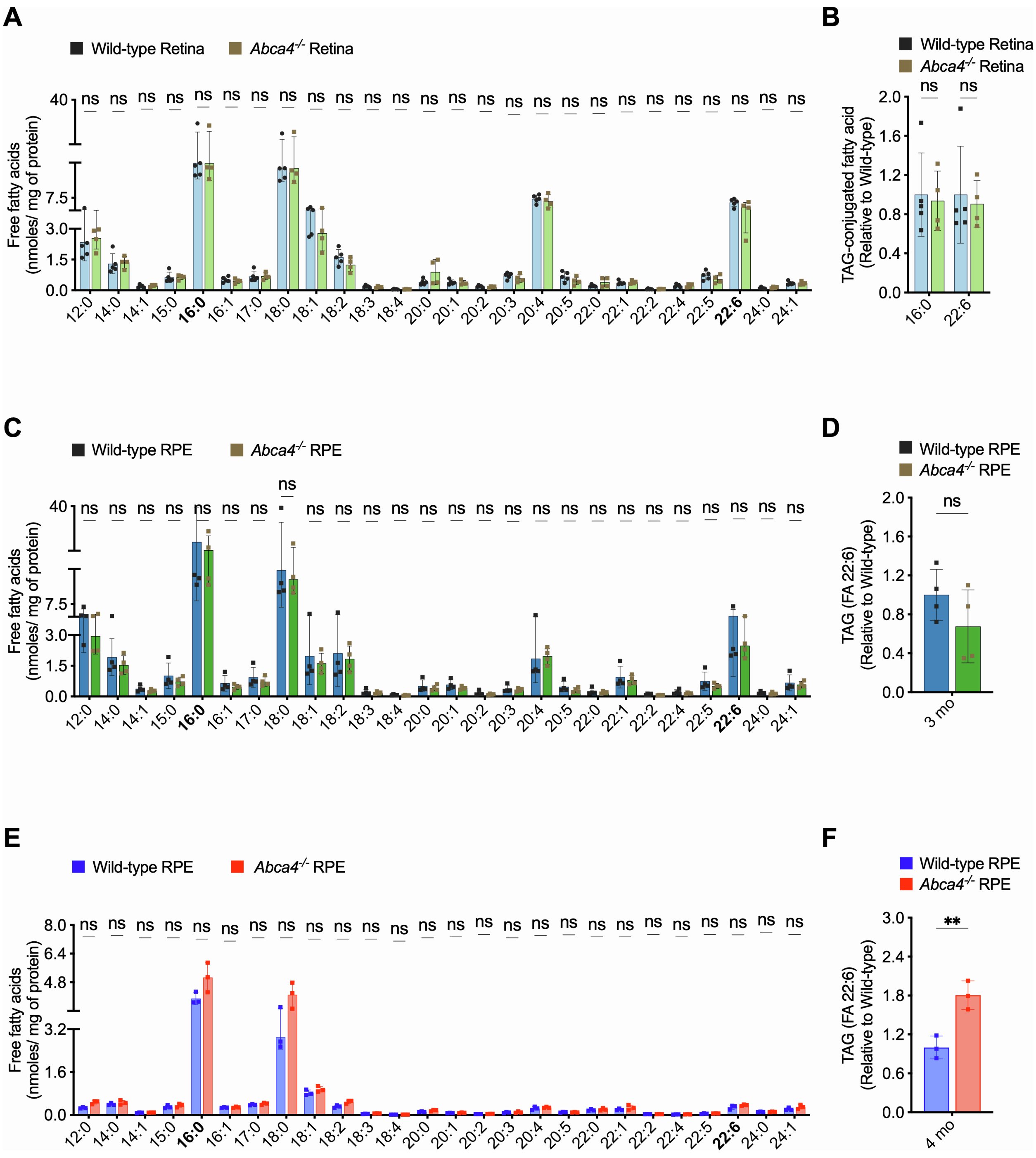

Suppl. Fig. S5 Free fatty acids profile in *Abca4*$^{-/-}$ and wild-type mice RPE. [A] Bar graph showing levels of free fatty acid species in the retina of 3-month-old wild-type (129/Sv) and *Abca4*$^{-/-}$ (129/Sv) mice. **[B]** Palmitic acid (FA 16:0) and DHA (FA 22:6) conjugated to TAG level in 3-month-old *Abca4*$^{-/-}$ (129/Sv) mouse retina, expressed relative to wild-type controls. **[C]** Levels of free fatty acid-species in RPE from 3-month-old wild-type (129/Sv) and *Abca4*$^{-/-}$ (129/Sv) mice.

[D] DHA conjugated to TAG level in RPE from 3-month-old *Abca4*$^{-/-}$ (129/Sv) mice, expressed relative to wild-type controls. **[E]** Levels of free fatty acid-species in RPE from 4-month-old wild-type (129/Sv) and *Abca4*$^{-/-}$ (129/Sv) mice. **[F]** DHA conjugated to TAG level in RPE from 4-month-old *Abca4*$^{-/-}$ (129/Sv) mice, expressed relative to wild-type controls. Data are presented as mean ± SD. Statistical significance was determined using unpaired two-tailed *t*-tests with Bonferroni correction for multiple comparisons; adjusted $^{*}p < 0.05$; $^{**}p < 0.01$; *ns* – not statistically significant.

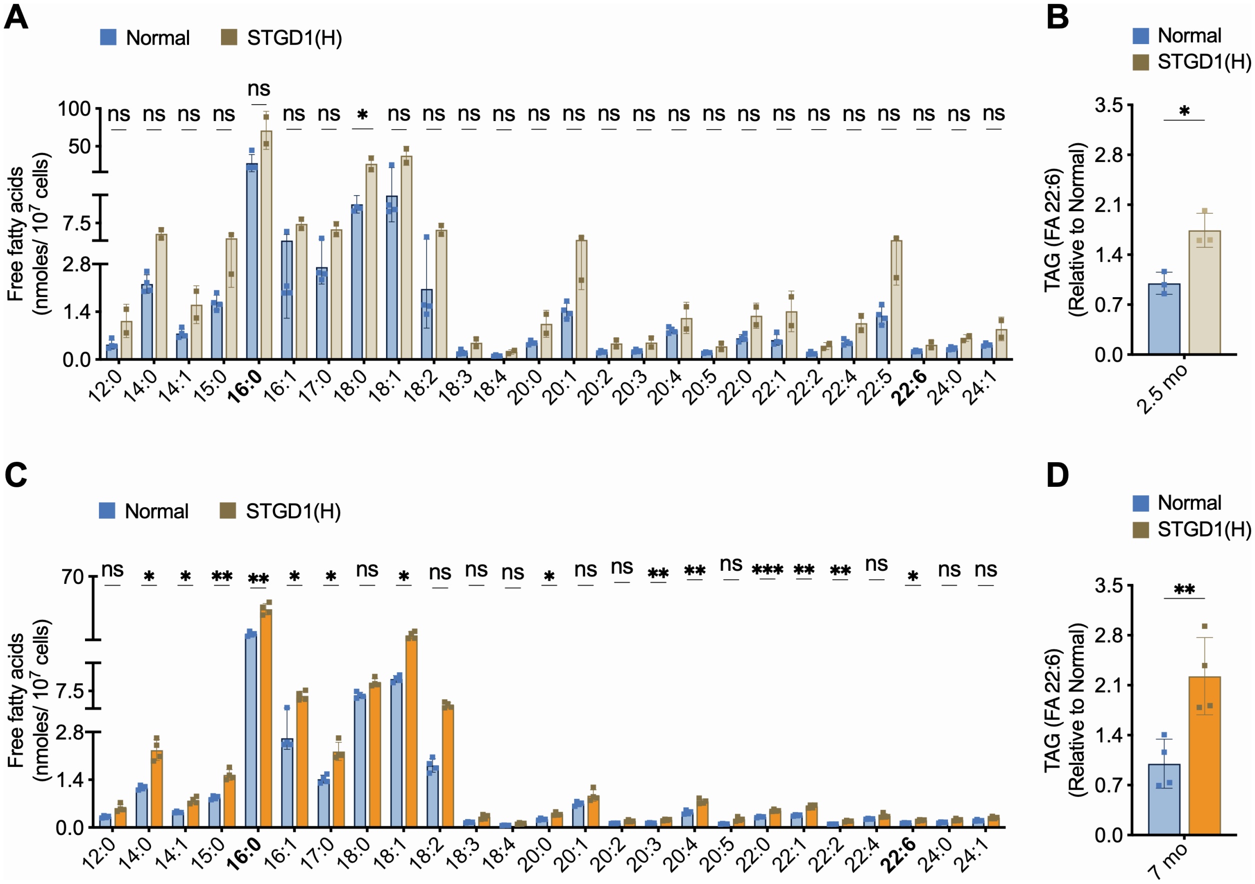

Suppl. Fig. S6 Free fatty acids profile in STGD1(H) and normal RPE. [A] Bar graph showing levels of free fatty acid-species in Normal and STGD1(H)-iPSC RPE cells grown for 2.5 months. **[B]** DHA conjugated to TAG level in 2.5-month RPE cultures, expressed as relative to Normal control. **[C]** Bar graph shows levels of free fatty acid-species in Normal and STGD1(H)-iPSC RPE cells cultured for 7 months. **[D]** DHA conjugated to TAG level in 7-month RPE cultures, expressed as relative to Normal control. Data are presented as mean ± SD. Statistical significance was determined using unpaired two-tailed *t*-tests with Bonferroni correction for multiple comparisons; adjusted $*p < 0.05$; $**p < 0.01$; $***p < 0.001$; *ns* – not statistically significant.

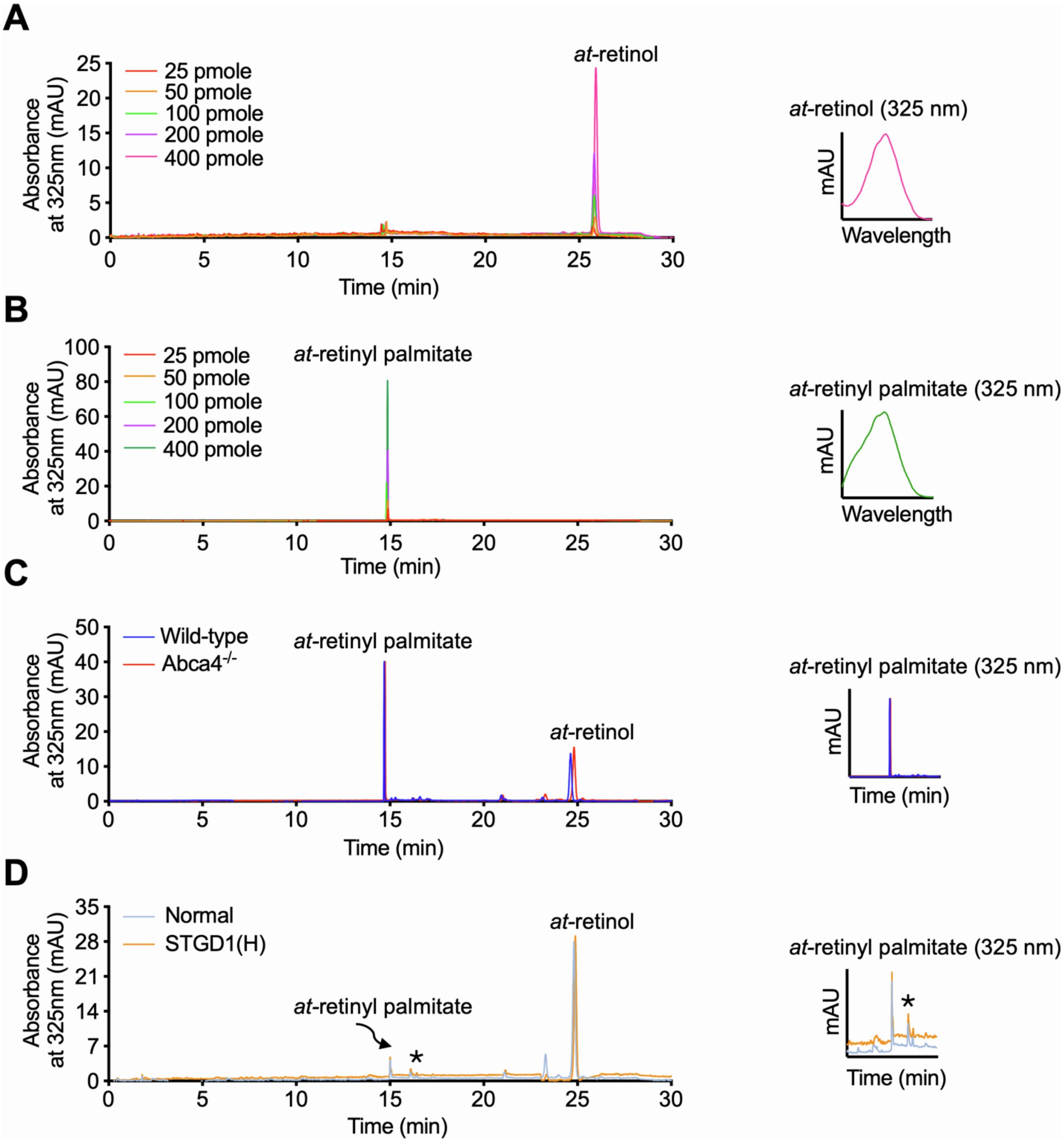

Suppl. Fig. S7 Representative HPLC chromatograms for LRAT assay in Fig.2E-H. Representative chromatograms at 325 nm of authentic retinoid standards (25, 50, 100, 200, and 400 pmoles) for all-*trans*-retinol (peak at ~25.6 min) in **[A]** and all-*trans*-retinyl palmitate (peak at ~14.8 min) in **[B]**; retinoid spectra are shown on the right. Representative chromatograms of LRAT activity using mouse RPE homogenates (wild-type (blue trace) and *Abca4*$^{-/-}$ (red trace)) in **[C]**

and iPSC RPE homogenates (normal, light-blue trace) and STGD1(H), orange trace) in **[D]**; zoom-in 12 to 18 min, *at*-RP peak co-eluting with authentic *at*-RP standard as shown in [B]; (*) asterisks indicate non-retinoid peak. Data for LRAT activity measured as *at*-RP formation using mouse and human RPE homogenates are shown in main Fig. 2E and Fig. 2H, respectively.

A

Protein Name	Short hand	Protein Name	Short hand
**Beta-oxidation pathway**		**Mitochondrial anion channels**	
Enoyl-CoA hydratase	ECHS1	Voltage-dependent anion-selective channel protein 1	VDAC1
3-hydroxyacyl-CoA dehydrogenase A	HADHA	Voltage-dependent anion-selective channel protein 2	VDAC2
3-hydroxyacyl-CoA dehydrogenase B	HADHB	**Oxidative phosphorylation pathway**	
**Carnitine Shuttle**		NADH dehydrogenase [ubiquinone] flavoprotein 1	NDUFV1
Carnitine O-palmitoyltransferase 1	CPT1A	NADH dehydrogenase [ubiquinone] flavoprotein 2	NDUFV2
Carnitine O-palmitoyltransferase 2	CPT2	Succinate dehydrogenase [ubiquinone] flavoprotein subunit	SDHA
**Glycolysis pathway**		Cytochrome b-c1 complex subunit Rieske	UQCRFS1
Hexokinase-1	HK1	Cytochrome b-c1 complex subunit 2	UQCRC2
Phosphoglucose isomerase	GPI	Cytochrome c oxidase subunit 5A	COX5A
Aldolase A	ALDOA	Cytochrome c oxidase subunit 5B	COX5B
Triosephosphate isomerase	TPI1	ATP synthase-coupling factor 6	ATP5PF
Glyceraldehyde-3-phosphate dehydrogenase	GAPDH	ATP synthase subunit g	ATP5MG
Phosphoglycerate kinase 1	PGK1	ATP synthase subunit f	ATP5MF
Phosphoglyceromutase 1	PGAM1	ATP synthase subunit O	ATP5PO
Aldo-enolase	ENO1	ATP synthase F(0) complex subunit B1	ATP5PB
Pyruvate kinase	PKM		

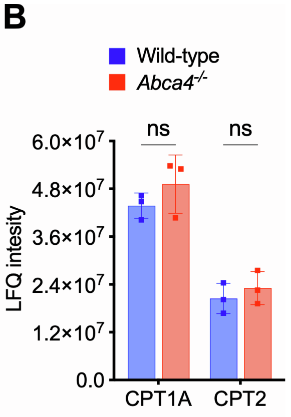

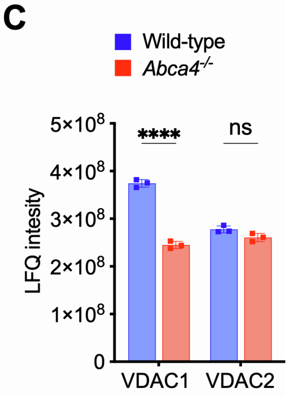

Suppl. Fig. S8 Proteomics data from *Abca4*$^{-/-}$ and wild-type mice RPE. [A] Metabolic pathway protein abbreviations used in Fig.5. **[B]** Carnitine shuttle and **[C]** Mitochondrial voltage-dependent anion channel (VDAC) protein levels in RPE from *Abca4*$^{-/-}$ mice (129/Sv). Data are presented as mean ± SD. Statistical significance was determined using unpaired two-tailed *t*-tests with Bonferroni correction for multiple comparisons; adjusted ****$p < 0.0001$; *ns* – not statistically significant.

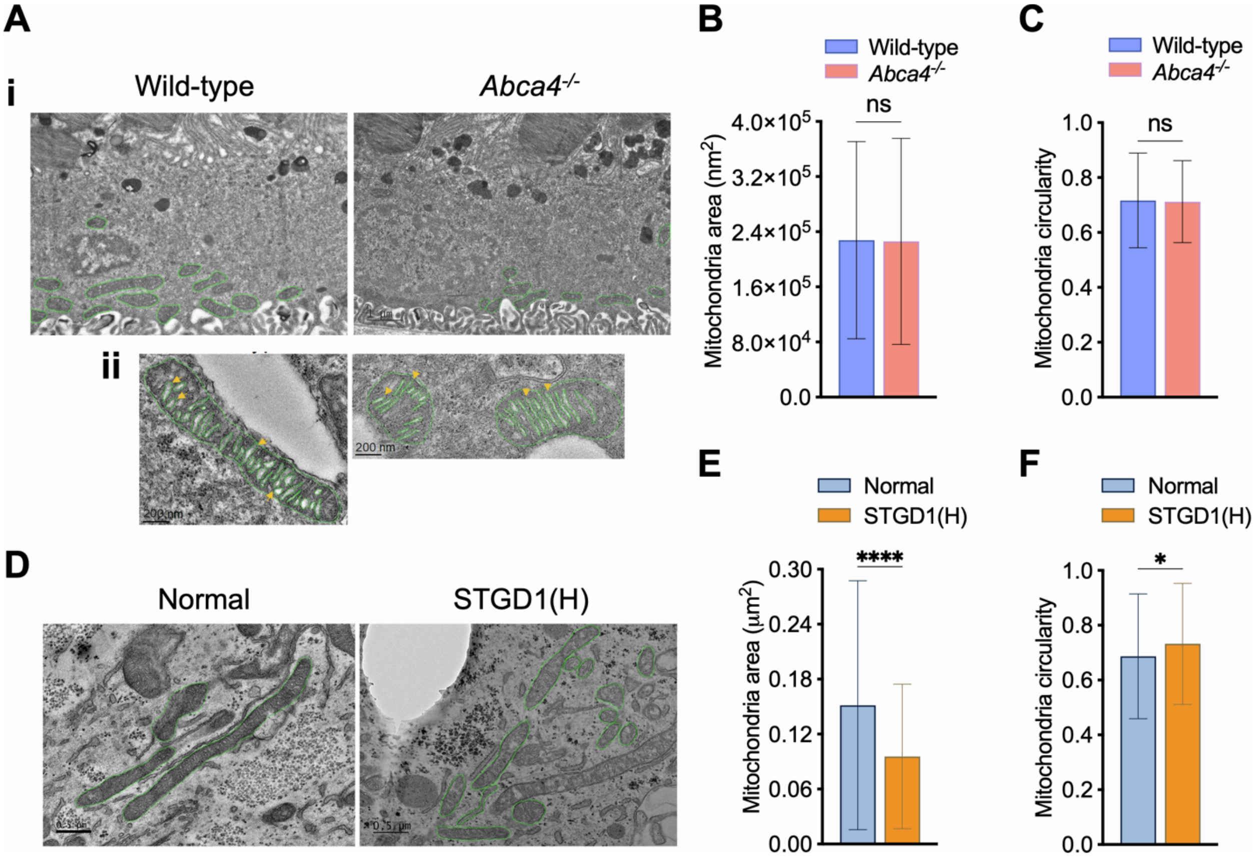

Suppl. Fig. S9 TEM image analysis of *Abca4*$^{-/-}$ mice and STGD1 patient RPE cells. [A] Representative transmission electron microscopy (TEM) images or RPE from6-month-old *Abca4*$^{-/-}$ vs. BALB/c wild-type mice. (i) mitochondria area is outlined in green (scale bar 1μm) and (ii) higher magnification view showing mitochondria and cristae area outlined in green (bottom, scale bar 200nm); yellow arrows highlight cristae circularity/curvature changes. Bar graphs show **[B]** mitochondria area and **[C]** mitochondria circularity quantified in *Abca4*$^{-/-}$ vs. wild-type RPE cells. **[D]** Representative TEM images of mitochondria and cristae (outlined in green) in Normal and STGD1(H) patient iPSC-RPE cultured for 6 months in culture (scale bar 0.5μm). Bar graphs show **[E]** mitochondria area and **[F]** mitochondria circularity quantified in STGD1(H) vs. Normal RPE cells (Extended analysis of Fig. 5). Data are presented as mean ± SD; unpaired *t*-test; *$p<0.05$, ****$p<0.0001$; *ns* – not statistically significant.

Table S1 Acronyms

Abbreviations	Full name
**POS**	Photoreceptor outer-segment
**ABCA4**	ATP-binding cassette, sub-family A, member 4
**RDHs**	Retinol dehydrogenase
**DGAT1**	Diacylglycerol O-acyltransferase-1
**LRAT**	Lecithin retinol acyltransferase
**RPE65**	Retinoid isomerase 65kDa
**GAPDH**	Glyceraldehyde-3-phosphate dehydrogenase
**OXPHOS**	Oxidative phosphorylation
**a*t*-RP**	all-*trans*-retinyl palmitate
**at-ROL**	all-*trans*-retinol
**a*t*-RAL**	all-*trans*-retinal
**11*c*-RAL**	*11-cis*-RAL
**TAG**	Triacylglycerol
**DAG**	Diacylglycerol
**FFA**	Free fatty acid
**PC**	Phosphatidylcholine
**PE**	Phosphatidylethanolamine
**LPC**	Lysophosphatidylcholine
**LPE**	Lysophosphatidylethanolamine
**CE**	Cholesterol ester
**PA**	Phosphatidic acid
**DHA**	Docosahexaenoic acid
